# Genetic Diversity and Population Structure of *Cylindrocarpon*-like Fungi Infecting Ginseng Roots in Northeast China

**DOI:** 10.3390/jof8080814

**Published:** 2022-08-02

**Authors:** Xiaohong Lu, Ximei Zhang, Xiaolin Jiao, Jianjun Hao, Shidong Li, Weiwei Gao

**Affiliations:** 1 Institute of Medicinal Plant Development, Chinese Academy of Medical Sciences and Peking Union Medical College, Beijing 100193, China; luxiaohong@caas.cn (X.L.); zhangximei2008@163.com (X.Z.); jiao_1110@163.com (X.J.); 2 Institute of Plant Protection, Chinese Academy of Agricultural Sciences, Beijing 100193, China; sdli@ippcaas.cn; 3 School of Food and Agriculture, University of Maine, Orono, ME 04469, USA; jianjun.hao1@maine.edu

**Keywords:** genetic differentiation, *Ilyonectia*, *Panax*, population structure, *histone H3*

## Abstract

Northeast China is well known for cultivating ginseng (*Panax ginseng* and *P. quinquefolius*). Ginseng roots are threatened by the infection of the most notorious *Cylindrocarpon*-like fungi (CLF), which are a complex containing important soilborne pathogens. Although the disease is economically important, little is known about the genetic diversity and population structure of the pathogenic CLF complex. This knowledge will help in developing effective disease management strategies. To conduct this study, diseased ginseng roots were collected from 12 regions representing the main ginseng-growing areas in Northeast China, and CLF were isolated. A total of 169 isolates with CLF anamorph were identified in two *Dactylonectria* species and six *Ilyonectria* species using morphological and molecular methods. Cross pathogenicity tests showed that all species were pathogenic to both *P. ginseng* and *P. quinquefolius*, and most of them had slightly stronger aggressiveness in *P. ginseng*. The analysis of partial sequences of the *Histone H3* gene generated a high level of genetic diversity and geographic differentiation. A total of 132 variable sites were detected in 169 sequences, which formed 20 haplotypes with a haplotype diversity of 0.824. Genetic differentiation was positively correlated with geographic distance. The geographic populations in the range of Changbai Mountain were distinctly discriminated from those in other non-Changbai Mountain populations. No significant genetic differentiation was found between ginseng hosts. *Cylindrocarpon*-like fungi causing ginseng root diseases are geographically correlated in the genetic distance in Northeast China and should be managed correspondingly.

## 1. Introduction

Asian ginseng (*Panax ginseng* M.) and American ginseng (*P. quinquefolius* L.) are two important medicinal herbs in the genus *Panax*, and they are mainly cultivated in the Northeastern region of China. Particularly, the region of Changbai Mountain has a long cultivation history of Asian ginseng. American ginseng has been planted in Northeast China since the 1980s. Notably, commercial growers commonly plant Asian ginseng and American ginseng on the same farm. More interestingly, they rotate the two species sequentially to avoid replant obstacles and fully utilize the farmland. Ginsengs are perennial plants, and their roots are usually harvested after four to six years of growth in the field. Such a long time of soil exposure makes them vulnerable to infection by soilborne pathogens.

In Northeast China, the most notorious disease of Asian ginseng is red-skin root, which is caused by a complex being consisting of 12 species of fungi [1]. It is common that the complex contains *Fusarium* spp. and *Rhexocercosporidium panacis*. In addition, seven species of CLF anamorphs, such as *Ilyonectria* species, take account for two-thirds of the total population. Similar to red-skin root of Asian ginseng, rusty root rot is another devastating disease and is also mostly caused by species with CLF anamorphs [2].

For American ginseng, Cylindrocarpon root rot and rusty root rot are the most important root diseases. Although Northeast China is an important ginseng-producing region, these diseases have not been well described. Cylindrocarpon root rot of American ginseng is also called disappearing root rot due to the symptoms of complete decay of diseased roots. It is mainly caused by *Cylindrocarpon* species, which have been well documented in Canada [3,4]. Rusty root rot of American ginseng can be caused by several *Fusarium* species and *R. panacis* [5,6]. In China, several *Fusarium* species, including *F. solani*, *F. oxysporum*, *F. dlamini* and *F. armeniacum* have been reported to cause disappearing root rot in American ginseng [7,8]. *Cylindrocarpon destructans*, *C. panacis*, *C. obtusisporum* and *C.* *panacicola* have been reported sporadically to cause rusty root rot [9,10]. Overall, fungi with CLF anamorphs are dominant pathogens causing root diseases in both Asian ginseng and American ginseng. It is not clear whether the pathogenic CLF differed in pathogenicity and virulence on both Asian ginseng and American ginseng.

It is a challenge to effectively manage such a pathogen complex in ginseng root disease control. Clarification of the population structure and host specificity of pathogens will be significant for making targeted disease management strategies. Geographic factors may affect the pathogen complex through temperature, humidity and other conditions. In addition, some CLF species not only infect *Panax* species but have a broad host range from herbaceous plants to woody plants, such as *Lilium* sp., *Vitis vinifera*, *Thymus* sp., *Quercus* spp., *Protea* sp. and *Leucospermum* spp. [11]. Genetic diversity of *C. destructans* from Asian ginseng in Korean has been associated with hosts based on RAPD finger printing or sequence analysis of nuclear ribosomal gene internal transcribed spacer (ITS) and mitochondrial small subunit (mt SSU) rDNA; however, in the study, only less than 20 isolates were investigated [12,13]. Although there are no genetic markers employed specifically in genetic study of CLF, several phylogenetic analyses of CLF showed that histone H3 (*his3*) gene had the highest resolution among several gene loci [1,11].

In the present study, our aims were to (i) identify the CLF isolates causing American ginseng root rot in Northeast China; (ii) determine the cross-pathogenicity between Asian ginseng and American ginseng; (iii) characterize the genetic structure of the CLF isolates from ginseng across the main growing regions in Northeast China; and (iv) investigate the geographic and host-origin effects on the population structure. Knowledge of the population genetics of CLF isolates would be important for developing sustainable and effective strategies for ginseng root disease control.

## 2. Materials and Methods

### 2.1. Isolation and Identification of CLF

**Figure 1:**
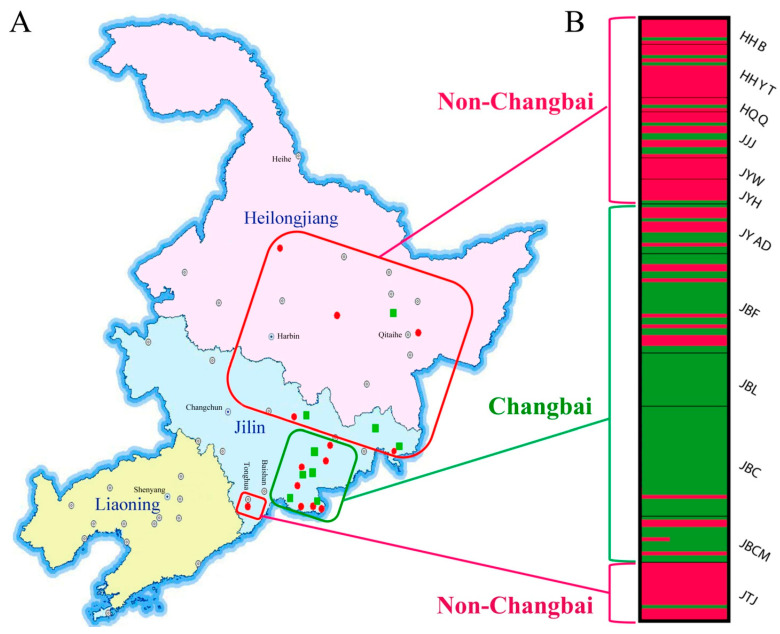
Population structure of *Cylindrocarpon*-like fungi from ginseng roots representing 22 locations in Northeast China. (**A**) Map showing sampling locations for *Panax ginseng* (red dot) and *P. quinquefolius* (green square). (**B**) Two clusters (*K* = 2) were identified from 12 populations according to the program STRUCTURE. Green rectangles represent all Changbai groups, and red rectangles represent non-Changbai group.

One hundred and sixty-nine isolates of CLF were obtained from diseased roots of Asian ginseng representing 13 locations and American ginseng representing 9 locations in Northeast China (Figure 1A and Supplementary Table S1). Isolates from every one or two nearby locations were treated as one geographic population, and a total of 169 isolates from 22 locations were grouped into 12 geographical populations (Table 1). Ninety-nine isolates from Asian ginseng with red-skin disease symptoms were previously obtained in 2012 and 2013 and have been identified by morphological and molecular methods in our previous study [1]. Seventy isolates from American ginseng showing rusty root or disappearing root symptoms were obtained in 2014 and identified as previously described [1]. Briefly, culture characteristics, including texture, density, color, growth front, trans parency and zonation, were visually examined. Morphological characteristics, including microconidia, macroconidia, conidiophores and chlamydospores, were observed under a microscope. For molecular identification, total genomic DNA was extracted from mycelia as previously described [1]. Partial sequences of the *his3* gene were amplified using the primer pair CYLH3F and CYLH3R [14]. PCR products were sequenced by Tianyihuiyuan Biotechnology Co., Ltd. (Beijing, China), assembled by using Chromas 1.5 (Technelysium Pty Ltd., Queensland, Australia), and edited with DNAMAN 6.0 (Lynnon BioSoft, Quebec, QC, Canada). A BLAST search of these sequences was performed using the nucleotide database of GenBank. Newly obtained unique sequences were deposited on the NCBI GenBank.

### 2.2. Pathogenicity Test

Randomly selected 1–3 isolates from each species and each host were evaluated for their pathogenicity on both Asian ginseng and American ginseng as previously described [1]. Briefly, conidial suspensions of CLF were drenched into pots containing potting soil with 2-year-old ginseng seedlings. From 10 to 14 replicated seedlings were inoculated for each isolate. Plants treated with sterile distilled water were used as a negative control (Mock). After 85 days of inoculation, disease severity was scored using a disease index ranging from 0 to 4, measured by the lesion area out of the whole root surface: 0, no symptoms; 1, ≤1/10; 2, >1/10 and ≤1/3; 3, >1/3 and ≤2/3; 4, >2/3. Pathogens were re-isolated from diseased roots and identified by morphological and molecular methods as described above.

### 2.3. Acquisition of Partial Sequences of Histone H3 Gene

Partial sequences of the *his3* gene of 99 isolates from *P. ginseng* were obtained previously [1], and sequences of 70 isolates from *P. quinquefolium* were obtained in this study. The information of some CLF isolates obtained from *Panax* spp. worldwide was collected for population structure analyses (Supplementary Table S2). The information of isolates in *I. robusta* and *I. mors-panacis* from other hosts were also collected for haplotype analyses (Supplementary Table S2).

### 2.4. DNA Polymorphism and Haplotype Analyses

Sequences of the *his3* gene were aligned using the MAFFT sequence alignment program v.6 [15]. The alignment was edited by using Bioedit v.7.0.4 for further analysis with molecular statistical programs. DNA polymorphism of *his3* gene sequences was determined by using DnaSP v. 6.12 [16].

DnaSP v. 6.12 was also used to generate the Arlequin haplotype data files [16]. Arlequin v. 3.5.1.2 was used to calculate the haplotype frequencies in populations and pairwise genetic differentiation coefficient (*F_ST_*) values [17]. *F_ST_* examines the extent of genetic differentiation between every two groups and takes values between zero and one. If *F_ST_* is less than 0.05, it means slight genetic differentiation; if *F_ST_* is more than 0.25, it means very high genetic differentiation. PopART v. 1.7 was used to calculate the relationships among haplotypes and to generate a visible TCS network with default settings [18,19].

### 2.5. Population Structure

Three methods were used to determine the population structure. Firstly, the STRUCTURE v.2.3.4 software was used to access the number of genetic clusters and estimated admixture, which implements a clustering algorithm based on Bayesian Monte Carlo Markov Chain approach to assign individuals into *K* distinct populations [20]. Searches with ancestral number of clusters ranging from *K* = 1 to 13 were performed in independent runs with 100,000 generations and a burn-in of 100,000. The number of genetically homogeneous clusters (*K*) was identified by following the method developed previously [21]. Secondly, Nei’s unbiased genetic distance was calculated based on gene frequency differences among all paired populations [22], and a principal coordinated analysis (PCoA) was run by GenAlEx6 [23]. Thirdly, an analysis of molecular variance (AMOVA) was performed to determine genetic differentiation using the GenALEx6 [23], which refers to the relative contribution among- and within-site components to the genetic variation.

### 2.6. Correlation between Genetic Variation and Geographic Separation

To determine whether genetic variation was correlated with geographic distance among CLF populations, the relationship between the genetic distance and the geographic distance was investigated by using a Mantel test implemented in GenALEx6 [23]. The pairwise Nei’s population genetic distances were compared to geographic distances between populations.

## 3. Results

### 3.1. Identification and Pathogenicity of CLF Isolates

Seventy CLF isolates were obtained from diseased American ginseng roots and classified into two genera *Ilyonectria* and *Dactylonectria* (Supplementary Table S1). Among them, 21 isolates were identified as *I.* communis, 17 isolates as *I. mors-panacis*, and 29 isolates as *I. robusta*. These isolates showed the same morphological characteristics and identical sequences of the *his3* gene as the ex-type isolates, respectively. The remaining three isolates were identical in sequences of the *his3* gene to isolate J711 from Asian ginseng. They were described as *Dactylonectria* sp. because they did not fall in any of the known species (Supplementary Table S1). All 169 CLF isolates belonging to two *Dactylonectria* species and six *Ilyonectria* species were collected (Table 2). *Ilyonectria communis*, *I. robusta* and *I. mors-panacis* were the top three most widely distributed species (Table 2). Eighty-eight *I. communis* isolates were collected from 10 out of 12 locations. Fifty isolates in *I. robusta* and 18 isolates of *I. mors-panacis* were obtained from nine and seven locations, respectively. Other species with frequencies of no more than five isolates were obtained from three locations at most. Most isolates (17 out of 18) in *I. mors-panacis* were collected from American ginseng, while isolates in *I. changbaiensis* and *I. qitaiheensis* were obtained from Asian ginseng only.

**Figure 2:**
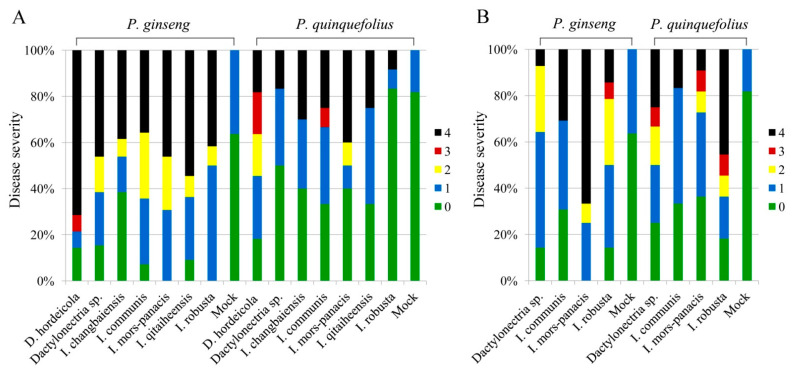
Disease severity of *Cylindrocarpon*-like fungi on *Panax ginseng* and *P. quinquefolius* roots. (**A**): Inoculation with isolates from *P. ginseng*; (**B**): Inoculation with isolates from *P. quinquefolius*. Disease symptoms were scored using a disease index ranging from 0 to 4, measured by the lesion area out of the whole root surface: 0, no symptoms; 1, ≤1/10; 2, >1/10 and ≤1/3; 3, >1/3 and ≤2/3; 4, >2/3.

Koch’s postulates were fulfilled for all *Cylindrocarpon*-like isolates tested on ginseng seedlings. No lesions were observed or minor lesions developed in the healthy control roots treated with sterile distilled water. All isolates were re-isolated from disease lesions and identified to the species inoculated initially, but no pathogenic fungi were isolated from mock roots. These isolates exhibited pathogenicity on both Asian ginseng and American ginseng, regardless of their original hosts (Supplementary Figures S1 and S2). Most isolates, especially isolates from *P. ginseng,* showed slightly higher virulence on Asian ginseng than on American ginseng (Figure 2).

### 3.2. Genetic Variation

A total of 132 variable sites were detected in 169 sequences of the *his3* gene with 468 bp (Table 3). The number of variable sites for each population ranged from 4 to 99 (Table 4). These polymorphic sites formed 20 haplotypes with a haplotype diversity of 0.824. The number of haplotypes for each population ranged from two to eight. The overall nucleotide diversity of 169 *Cylindrocarpon*-like isolates was 0.045. The highest nucleotide diversity (0.059) was observed in the JBCM population, while the lowest nucleotide diversity (0.004) was observed in the JYW population (Table 4).

**Figure 3:**
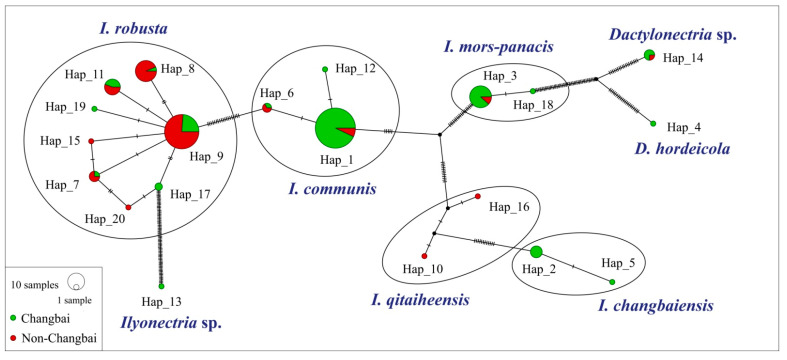
TCS haplotype network generated for *his3* gene sequences representing two geographical groups of *Cylindrocarpon*-like fungi. The size of the circle indicates the frequency of haplotype in the population. Hatch marks indicate the number of mutations.

A TCS network of the CLF isolates was constructed (Figure 3). Most haplotypes within one species were closely related and separated by one to three mutations. Hap_1 representing a genotype of *I. communis* was the most commonly shared haplotype being present in 57 sequences. Hap_9 representing a genotype of *I. robusta* was the second most commonly shared haplotype being present in 42 sequences. Species *I. robusta* had the highest number (eight) of haplotypes with 1–3 mutations. Species *I. communis* had only three haplotypes, although it was widely distributed in 10 locations (Table 2 and Figure 3).

The *F_ST_* values between populations ranged from −0.0967 to 0.731, and more than half of *F_ST_* values showed significant differentiation (Table 5). The highest *F_ST_* value (0.731) with significant differentiation was observed between the JTJ and JBL populations, and the lowest *F_ST_* value (−0.097) without significant differentiation was between JYH and HHB.

### 3.3. Population Structure

STRUCTURE analyses revealed two clusters of CLF isolates. The model with *K* = 2 had both the highest likelihood values and Evanno’s Δ*K* index, which were significantly higher than other clusters. At *K* = 2, the five populations in the Changbai group were separated from non-Changbai populations (Figure 1B).

**Figure 4:**
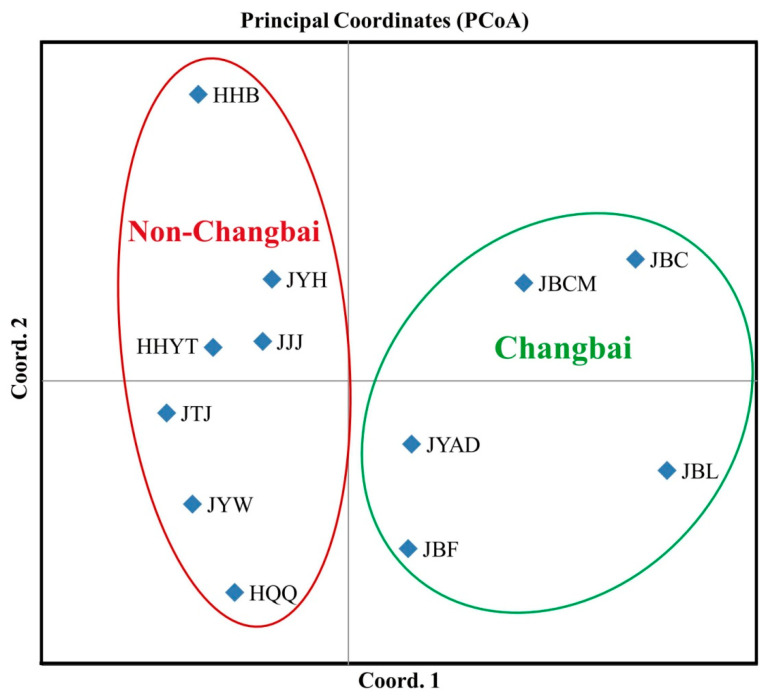
Principal coordinate analysis of 12 populations of *Cylindrocarpon*-like fungi based on Nei’s genetic distance by using GenAlEx. Axes 1 and 2 of the PCoA accounted for 85.38% and 7.05% of the total genetic variation. A green ellipse indicates all Changbai populations, and red ellipse indicates non-Changbai populations.

A similar clustering pattern was obtained by PCoA. Axes 1 and 2 of the PCoA accounted for 85.38% and 7.05% of the total genetic variation (Figure 4). PCoA also indicated that the Changbai populations were grouped in one cluster in the right quadrants of the first principal coordinate, and the rest of the seven non-Changbai populations from other locations clustered in the left quadrants (Figure 4).

AMOVA also revealed significant variation among geographic regions (Changbai and non-Changbai), accounting for 36% of the total genetic variation (Table 6). Among individual isolates within populations exhibited 59% of the genetic variation, while only 5% of the genetic variation was attributed to variations among populations. All the sources of variation were significant (*p* = 0.01).

The Changbai group had more DNA variable sites than other populations (Table 4). In most cases, *F_ST_* values were consistently higher between pairs of two different geographic groups (Changbai and non-Changbai) than between pairs of the same geographic groups (Table 5). Correspondingly, the Changbai group had more haplotypes than the non-Changbai group (Table 4 and Figure 3). Among these haplotypes, there were eight haplotypes exclusively distributed in the Changbai group and four haplotypes exclusively distributed in the non-Changbai group (Figure 3).

### 3.4. Population Differentiation

**Figure 5:**
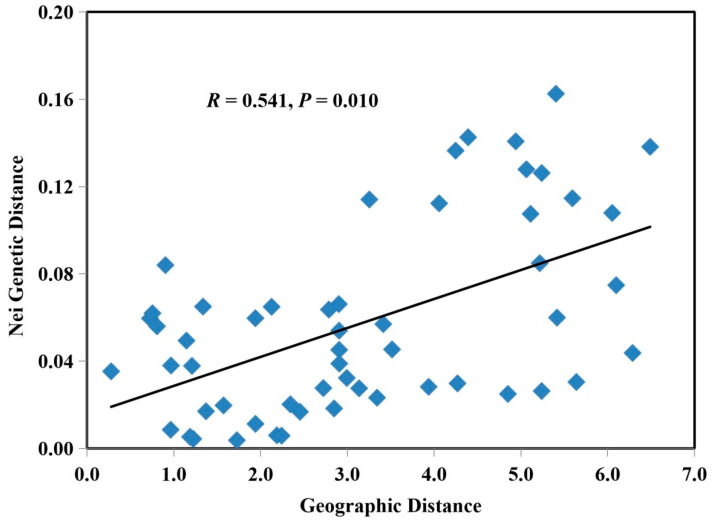
Correlation between genetic differentiation based on Nei’s genetic distance and geographic distance among 11 populations of *Cylindrocarpon*-like isolates by using Mantel test with GenAlEx.

The Mantel test showed that there was no correlation between geographic distance and genetic differentiation among all 12 geographic populations (Supplementary Figure S3). However, further analyses on 11 geographic populations without JTJ showed that geographic distance and genetic differentiation among populations were positively correlated (*R* = 0.541, *p* = 0.010, Figure 5). Population genetics of CLF isolates had a high differentiation between two geographic groups (*F_ST_* = 0.361, *p* < 0.001), but exhibited slight differentiation between two host groups (*F_ST_* = 0.046, *p* < 0.01). No significant differentiation was found between populations from two different hosts, *P. ginseng* and *P. quinquefolius,* by using STRUCTURE analysis (Supplementary Figure S4). Haplotypes exclusively isolated from *P. ginseng* were genetically far away from other haplotypes, such as haplotypes belonging to *I. changbaiensis* and *I. qitaiheensis* (Supplementary Figure S5).

PCoA of worldwide CLF isolates from *Panax* spp. showed a significant population differentiation (Supplementary Figure S6). Axes 1 and 2 of the PCoA accounted for 83.21% and 11.96% of the total genetic variation (Supplementary Figure S6). PCoA also indicated that the Changbai population was located in the first principal coordinate and far away from other populations. Other populations including isolates from this study and the Northeast population including isolates from Genbank were closely related. Yunnan, Shandong and Canadian populations including mostly *I. mors-panacis* were nearby each other. Among them, Shandong and Canadian populations with the same host *P. quinquefolius* were closely neighbored (Supplementary Figure S6).

Among these CLF species, *I. robusta* from 11 locations exhibited the most diversity and belonged to eight haplotypes (Figure 3). In further analyses of *I. robusta* sequences downloaded from the GenBank, 15 haplotypes were found from worldwide 15 host genera or habitats (Supplementary Figure S7). Haplotype 5 was the biggest group originating from six continents and nine host genera or habitats. In contrast to *I. robusta*, *I. mors-panacis* exhibited a low diversity and narrow host range (Supplementary Figure S8). Unique haplotypes were formed in *P. notoginseng* and *P. quinquefolius* populations but not from *P. ginseng*. Correspondingly, these unique haplotypes were exclusively from Yunnan, Shandong and Canada, which are production areas of *P. notoginseng* and *P. quinquefolius,* respectively.

## 4. Discussion

We provided a whole picture of the pathogen complex of CLF populations associated with ginseng root diseases in Northeast China by analyzing 169 isolates. Among the CLF species, *I. robusta*, *I. communis* and *I. mors-panacis* are the top three most frequently isolated species causing ginseng root diseases in Northeast China, which was also supported by previous studies [1,2]. Although *I. robusta* and *I. communis* were more frequently isolated than *I. mors-panacis*, they were less virulent than *I. mors-panacis*. *Ilyonectria mors-panacis* usually causes root rot on ginseng and was a highly virulent CLF species shown in this and other studies [24,25,26]. *Ilyonectria communis* is widely distributed in Northeast China, and its host range should be addressed in future research. In addition to *Ilyonectria* species causing ginseng root diseases described in the present study, *I. crassa* and *I. panacis* have been reported to cause root diseases in American ginseng in Canada [11].

We have provided the first in-depth assessment of the genetic structure of CLF populations associated with ginseng in Northeast China, which were clustered into two distinct genetic groups. The two groups were discriminated against by Changbai Mountain. The Changbai populations generated more haplotypes and higher nucleotide diversity than the other populations. The high and significant pairwise *F_ST_* value between two geographical groups and the genetic variation contributed by geographical regions also supported the two distinct genetic clusters. This could be due to a similar climate and longer ginseng growing history along the range of Changbai Mountain than in other areas in Northeast China. The Changbai group was even distinctly different from all other groups, including worldwide isolates. The retrieved Northeast group was close to the non-Changbai group of this study. This provides another evidence for the Changbai group with unique genetic populations.

We found a significantly positive correlation between genetic distance and geographic distance among CLF populations. This indicated that geographic and climatic factors contributed to the overall genetic differentiation in CLF populations associated with ginseng plantations. A similar result has been found in *I. liriodendri* causing black foot disease in New Zealand vineyards in a previous study [27]. Besides the local climate, the soilborne *Ilyonectria* species are soilborne and limited for long-distance spread, and their reproduction is restricted locally. Although seedling transportation can cause a long-distance spread of soilborne pathogens, the current data implied this was not a main contributing factor.

There was no tight association of CLF populations with their hosts *P. ginseng* and *P. quinquefolius* in Northeast China, based on STRUCTURE analysis. The conclusion could have been strengthened if the genetic relationships among these host populations were examined by using discriminant analysis of principal components and Bruvo genetic distance [28]. However, CLF isolates showed cross-pathogenicity in these two hosts, indicating their low host specificity. Interestingly, we found most CLF species had a higher virulence on *P. ginseng* than on *P. quinquefolius*. This partially explains why it is practicable to plant *P. quinquefolius* following harvesting of *P. ginseng* to avoid replant diseases in Northeast China. The species *I. robusta* has a broad host range, including woody plants and herbs [11] and contains a higher genetic diversity than *I. mors-panacis*, which has a narrow host range, suggesting that the genetics of the pathogen complex could be affected by alternative hosts. Given that various plant hosts are around ginseng fields in Northeast China, host shifts may be frequent. This could increase the possibility that *I. robusta* on nearby hosts could mate, which could increase the genetic differentiation of the *I. robusta* population.

Sexual reproduction should be another indispensable factor significantly affecting genetic differentiation. The teleomorph has been observed in vitro on *I. robusta* [1,11] but unknown in *I. communis* and *I. mors-panacis* [11]. Although *I. communis* had a high frequency than *I. robusta* in the present study, more haplotypes were observed in *I. robusta* than *I. communis*. It suggested that sexual reproduction may accelerate the genetic differentiation of *I. robusta*.

This is the first time of using the *his3* gene sequence as a genetic marker in studying the population structure of CLF. Rich sequence data of *his3* in the GenBank allowed us to compare the worldwide populations of *I. robusta* and *I. mors-panacis* with our isolates. A partial sequence of the *his3* gene with 468 bp generated strong differentiation and a high level of genetic diversity in 169 isolates, suggesting the *his3* gene is informative for population genetic studies in CLF. This is consistent with previous phylogenetic research that the *his3* gene has the highest resolution among the multi loci [1,11], which provided the same resolution as four genes combined [29,30,31]. The *his3* marker is not only essential to delineate CLF isolates at species level but also generate finer resolutions of the population within a species. It is common in genetic diversity studies of plants and animals to use *COI* gene, but it is rare to use one gene only in fungal genetic diversity research [32,33]. We strongly recommend providing *his3* gene sequences in taxonomy and phylogenetic studies of CLF. This method is worthy of application in the genetic study of CLF.

## 5. Conclusions

Population structure and genetic diversity of CLF causing root diseases in *P. ginseng* and *P. quinquefolius* were determined in Northeast China. Genetic differentiation of CLF was positively correlated with geographic distance. Geographic populations were found to be in two clusters, which were discriminated by Changbai Mountain. This result will advance the study of population genetics of CLF in order to better understand the impacts of geo-climate and hosts, as well as facilitate the development of effective disease management strategies for the pathogen complex.

## Figures and Tables

**Table 1:** *Cylindrocarpon*-like fungal populations analyzed in this study.

Population Code	Location			Host		Number of Isolates
	Province, City, County, Town	Longitude (East)	Latitude (North)	*Panax ginseng*	*P. quinquefolius*	
HHB	Heilongjiang, Heihe, Beian	47.77	126.78	7	0	7
HHYT	Heilongjiang, Harbin, Yilan and Tonghe	46.32	129.56	2	13	15
HQQ	Heilongjiang, Qitaihe, Qiezihe	45.75	131.19	4	0	4
JBC	Jilin, Baishan, Changbai	41.52	127.46	31	0	31
JBCM	Jilin, Baishan, Changbai, Malugou	41.44	128.21	7	6	13
JBF	Jilin, Baishan, Fusong	42.42	127.54	12	16	28
JBL	Jilin, Baishan, Linjiang	41.74	127.29	0	15	15
JJJ	Jilin, Jilin, Jiaohe	43.63	127.73	2	11	13
JTJ	Jilin, Tonghua, Jian	41.51	125.88	16	0	16
JYAD	Jilin, Yanbian, Antu and Dunhua	42.58	128.33	12	2	14
JYH	Jilin, Yanbian, Hunchun	43.42	129.66	6	1	7
JYW	Jilin, Yanbian, Wangqing	42.93	130.49	0	6	6
Total				99	70	169

**Table 2:** *Cylindrocarpon*-like fungi species identified.

Population Code	*D. hordeicola*	*Dactylonectria* sp.	*I. changbaiensis*	*I. communis*	*I. mors-panacis*	*I. qitaiheensis*	*I.robusta*	*Ilyonectria* sp.
JBC	1	0	5	23	1	0	1	0
JBCM	0	0	0	10	2	0		1
JBF	0	2	0	17	6	0	3	0
JBL	0	0	0	10	5	0		0
JYAD	0	1	0	7	2	0	4	0
Changbai-Total	1	3	5	67	16	0	8	1
HHB	0	0	0	7	0	0	0	0
HHYT	0	0	0	2	0	0	13	0
HQQ	0	0	0	1	0	1	2	0
JJJ	0	1	0	7	1	0	4	0
JTJ	0	0	0	0	0	1	15	0
JYH	0	0	0	4	1	0	2	0
JYW	0	0	0	0	0	2	6	0
Non-Changbai-Total	0	1	0	21	2	4	42	0
Total	1	4	5	88	18	2	50	1

**Table 3:** DNA polymorphisms of *Cylindrocarpon*-like fungi based on partial sequences of the *his3* gene.

DNA Polymorphism Parameter	*his3*
Number of sequences	169
Selected region analyzed	1–468
Number of variable sites, S	132
Number of mutation, Eta	138
Number of haplotypes, h	20
Haplotype diversity, Hd	0.824
Nucleotide diversity, Pi	0.045
Theta from S	0.054
Theta from Pi	0.047
Number of nucleotide differences, k	18.374

**Table 4:** DNA polymorphisms for *Cylindrocarpon*-like fungi based on partial of *his3* gene sequences.

Population	No. of Sequences	No. of Variable Sites	No. of Haplotypes	Haplotype Diversity	Nucleotide Diversity
JBC	31	99	7	0.527	0.033
JBCM	13	97	5	0.705	0.059
JBF	28	87	8	0.796	0.056
JBL	15	26	3	0.705	0.027
JYAD	14	87	6	0.857	0.057
Changbai-Total	101	130	16	0.693	0.047
HHB	7	25	2	0.286	0.016
HHYT	15	27	4	0.371	0.015
HQQ	4	40	3	0.833	0.045
JJJ	13	87	7	0.846	0.052
JTJ	16	38	4	0.35	0.011
JYH	7	40	5	0.905	0.027
JYW	6	4	3	0.733	0.004
Non-Changbai-Total	68	97	12	0.727	0.023
Total	169	132	20	0.824	0.045

**Table 5:** Pairwise *F_ST_* values among 12 populations of *Cylindrocarpon*-like isolates.

Population	JBC	JBCM	JBF	JBL	JYAD	HHB	HHYT	HQQ	JJJ	JTJ	JYH
JBCM	0.079 *										
JBF	0.128 ***	−0.031									
JBL	0.097 *	0.060	0.082								
JYAD	0.257 ***	0.010	−0.007	0.250 ***							
HHB	0.524 ***	0.260 **	0.219 **	0.6314 ***	0.056						
HHYT	0.577 ***	0.373 ***	0.310 ***	0.675 ***	0.175 **	0.098 *					
HQQ	0.420 ***	0.138	0.124	0.526 ***	−0.036	−0.079	0.133				
JJJ	0.339 ***	0.081	0.057	0.366 ***	−0.059	−0.021	0.123 *	−0.083			
JTJ	0.623 ***	0.434 **	0.351 ***	0.731 ***	0.212 ***	−0.052	0.196 ***	0.041	0.119		
JYH	0.530 ***	0.252 ***	0.205 **	0.601 ***	0.039	−0.097	0.069	−0.087	−0.022	−0.018	
JYW	0.617 ***	0.383 **	0.324 **	0.729 ***	0.170	0.005	0.066	0.097	0.089	−0.019	−0.033

*, 0.01 ≤ *p* < 0.05; **, 0.001 ≤ *p* < 0.01; ***, *p* < 0.001.

**Table 6:** Analysis of molecular variance (AMOVA) within and among 12 populations of *Cylindrocarpon*-like fungi.

Source *	df	SS	MS	Estimated Variance	Percentage	Stat	Value	*p*
Among regions	1	568.731	568.731	6.678	36%	PhiRT	0.358	0.010
Among populations	10	233.123	23.312	0.915	5%	PhiRR	0.077	0.010
Within populations	157	1733.122	11.039	11.039	59%	PhiPT	0.408	0.010
Total	168	2534.976		18.632	100%			

* The 169 *Cylindrocarpon*-like fungal isolates were grouped in two regions (Changbai and Non-Changbai) and 12 populations. df—degree of freedom; SS—sum of squared observations; MS—mean of squared observations; PhiRT—proportion of the total genetic variance between regions; PhiPR—proportion of the total genetic variance among populations within a region; PhiPT—proportion of the total genetic variance among individuals within a population.

## Data Availability

All data are included in the main text and Supplementary Materials online.

## Supplementary Materials

The following supporting information can be downloaded at: https://www.mdpi.com/article/10.3390/jof8080814/s1, Table S1: CLF isolates obtained from *Panax ginseng* or *P. quinquefolius* in Northeast China. Table S2: CLF isolates used in worldwide genetic diversity and population structure analyses. Figure S1: Pathogenicity test of CLF isolates obtained from *Panax ginseng* roots. Figure S2: Pathogenicity test of CLF isolates obtained from *P. quinquefolius* roots. Figure S3: Correlation between genetic differentiation based on Nei’s genetic distance and geographic distance among 12 populations of CLF isolates by using Mantel test with GenAlEx. Figure S4: Population structure of CLF isolates analyzed by using STRUCTURE program. Figure S5: TCS haplotype network based on partial sequence of the *his3* gene representing two populations of CLF isolates from *Panax ginseng* (blue) and *P. quinquefolius* (yellow). Figure S6: Principal coordinate analysis of 6 populations of CLF isolates from *Panax* species worldwide based on Nei’s genetic distance by using GenAlEx. Figure S7: TCS haplotype network based on partial sequence of the *his3* gene representing worldwide *Ilyonectria robusta* isolates. Figure S8: TCS haplotype network generated based on partial sequence of the *his3* gene representing worldwide *Ilyonectria mors-panacis* isolates.
