# Supplementary material for: Genetic Diversity and Population Structure of Cylindrocarpon-like Fungi Infecting Ginseng Roots in Northeast China
Source: J Fungi (Basel). 2022 Aug 2;8(8):814. doi: 10.3390/jof8080814 (PMC9410487; doi:10.3390/jof8080814)
Supplement: Supplementary file 1 [file jof-08-00814-s001.zip › jof-1805294-supplementary.pdf]

Supplementary Materials for

Genetic diversity and population structure of *Cylindrocarpon*-like fungi infecting ginseng roots in Northeast China

Figure S1 to S8 & Table S1 and S2

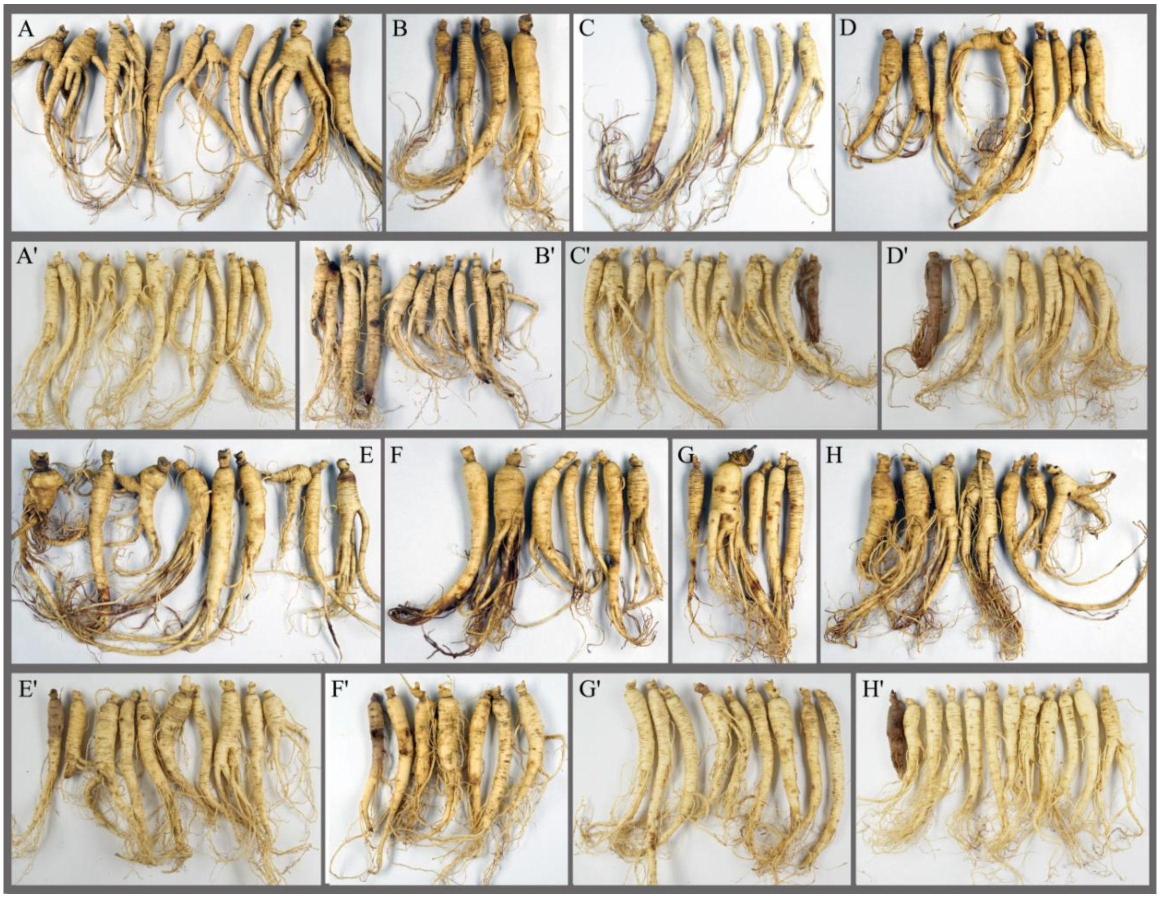

Figure S1. Pathogenicity test of CLF isolates obtained from *Panax ginseng* roots. A to H: virulence on *P. ginseng*, A' to H': virulence on *P. quinquefolius*; A & A: mock; B & B': *Dactylonectria hordeicola*; C & C': *Dactylonectria* sp.; D & D': *Ilyonectria changbaiensis*; E & E': *I. communis*; F & F': *I. mors-panacis*; G & G': *I. qitaiheensis*; H & H': *I. robusta*.

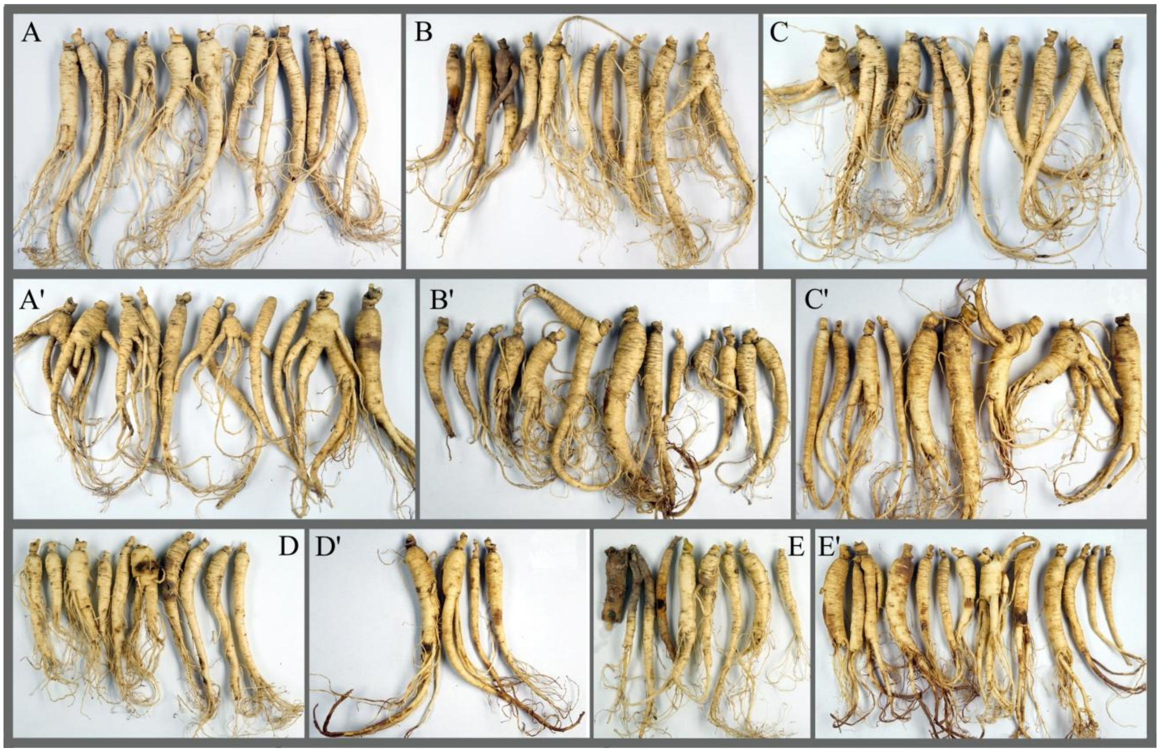

Figure S2. Pathogenicity test of CLF isolates obtained from *P. quinquefolius* roots. A to H: virulence on *P. quinquefolius*, A' to H': virulence on *P. ginseng*; A & A': mock; B & B': *Dactylonectria* sp.; C & C': *Ilyonectria communis*; D & D': *I. mors-panacis*; E & E': *I. robusta*.

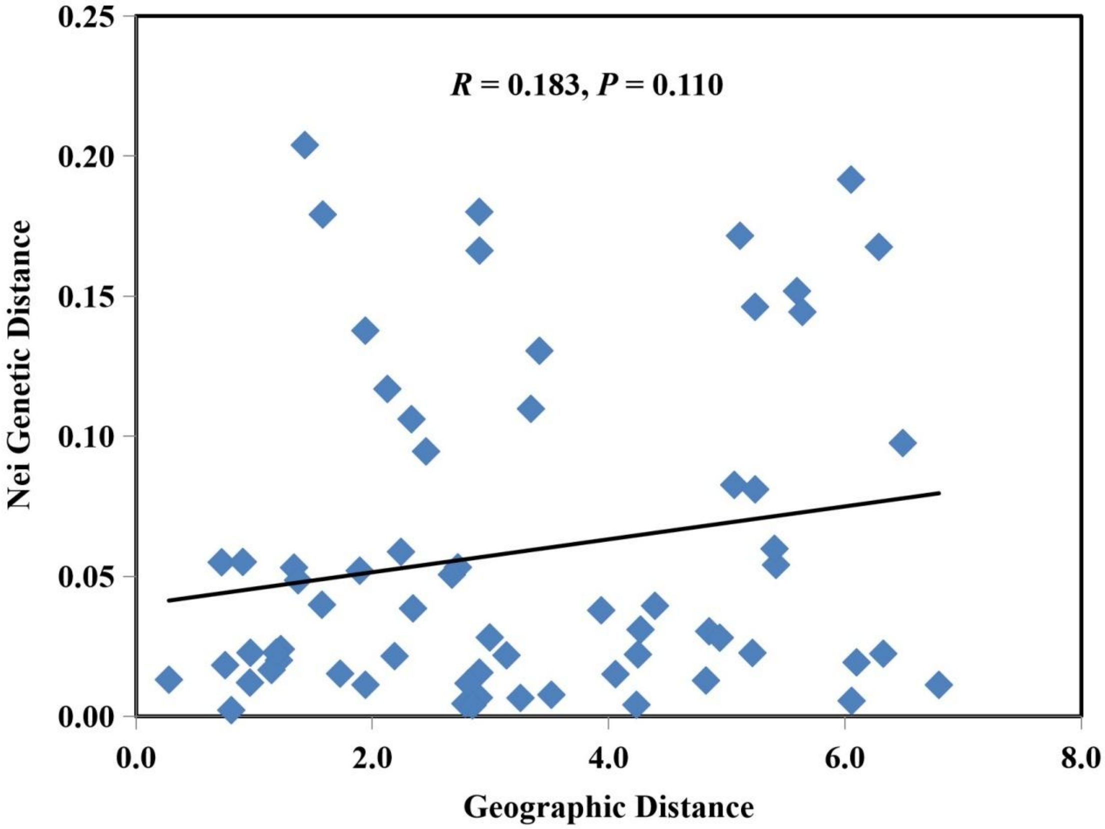

Figure S3. Correlation between genetic differentiation based on Nei's genetic distance and geographic distance among 12 populations of CLF isolates by using Mantel test with GenAlEx.

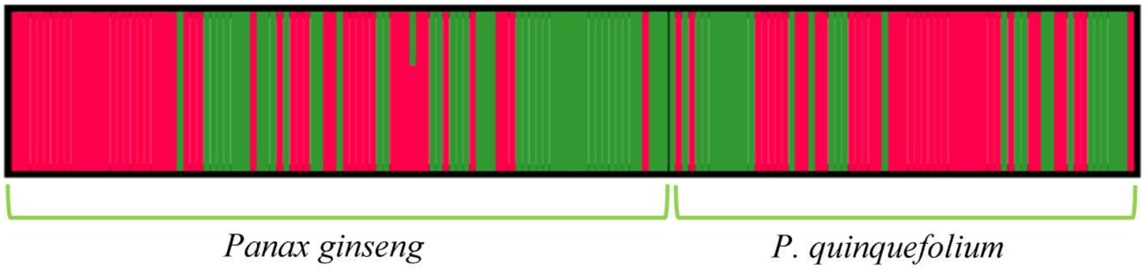

Figure S4. Population structure of CLF isolates analyzed by using STRUCTURE program. Two clusters (K = 2) were identified from two populations sampled from *Panax ginseng* and *P. quinquefolius* respectively.

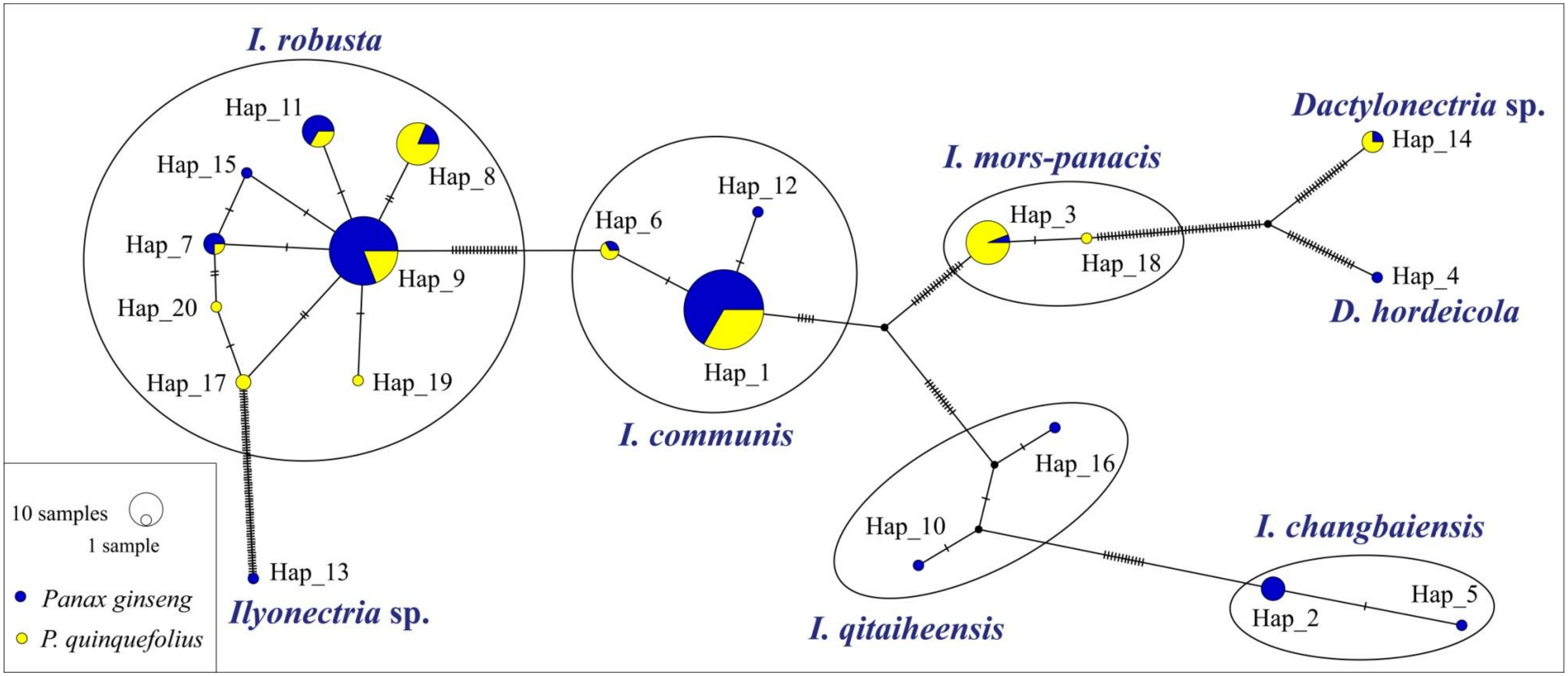

Figure S5. TCS haplotype network based on partial sequence of the *his3* gene representing two populations of CLF isolates from *Panax ginseng* (blue) and *P. quinquefolius* (yellow). The size of the circle represents the haplotype frequencies in populations. Hatch marks indicate the number of mutations.

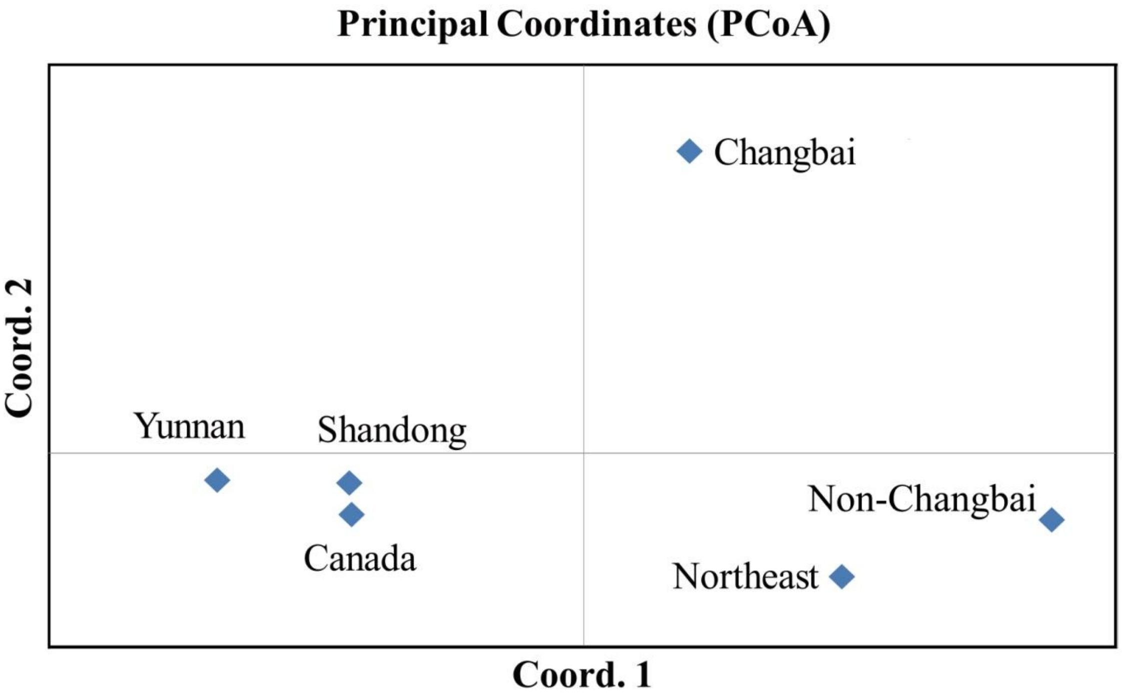

Figure S6. Principal coordinate analysis of 6 populations of CLF isolates from *Panax* species worldwide based on Nei's genetic distance by using GenAlEx. Axes 1 and 2 of the PCoA accounted for 83.21% and 11.96% of the total genetic variation.

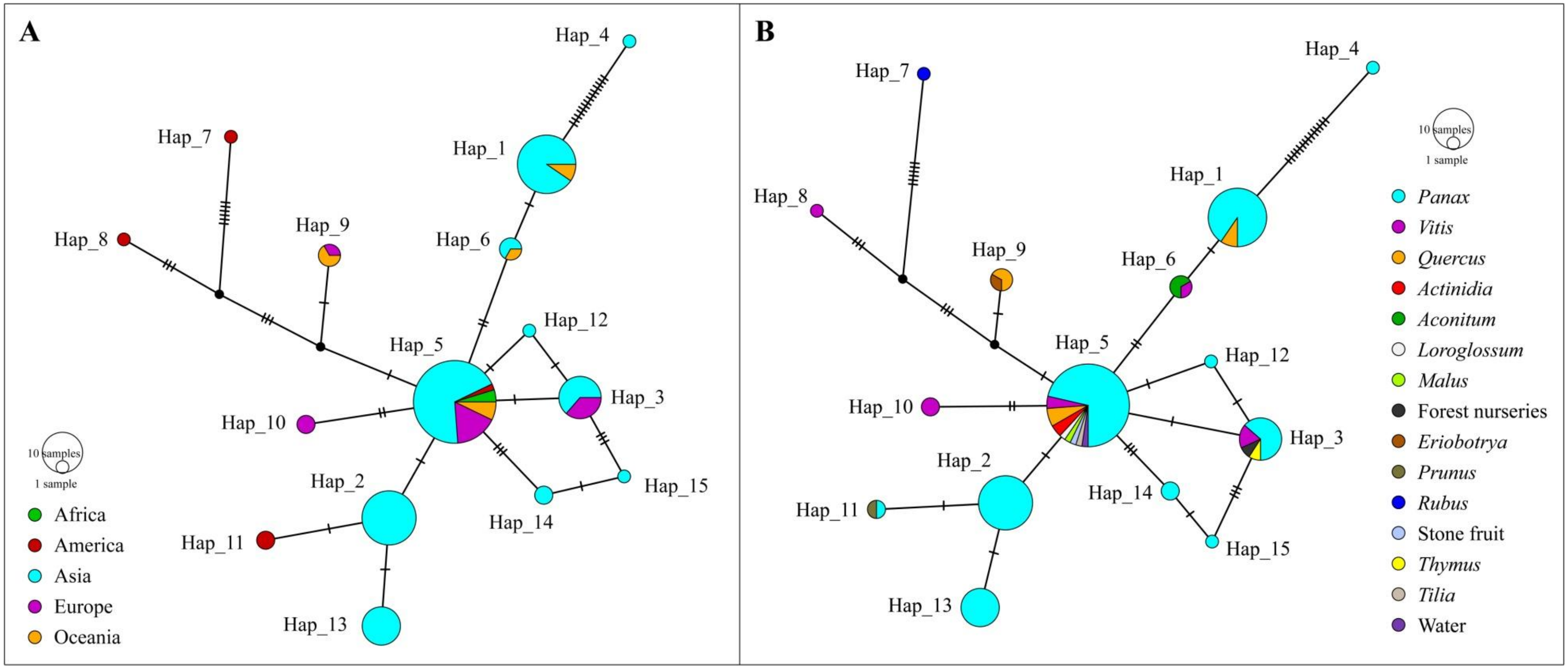

Figure S7. TCS haplotype network based on partial sequence of the *his3* gene representing worldwide *Ilyonectria robusta* isolates. A: geographical populations; B: populations divided based on hosts. The size of the circle represents the haplotype frequencies in populations. Hatch marks indicate the number of mutations.

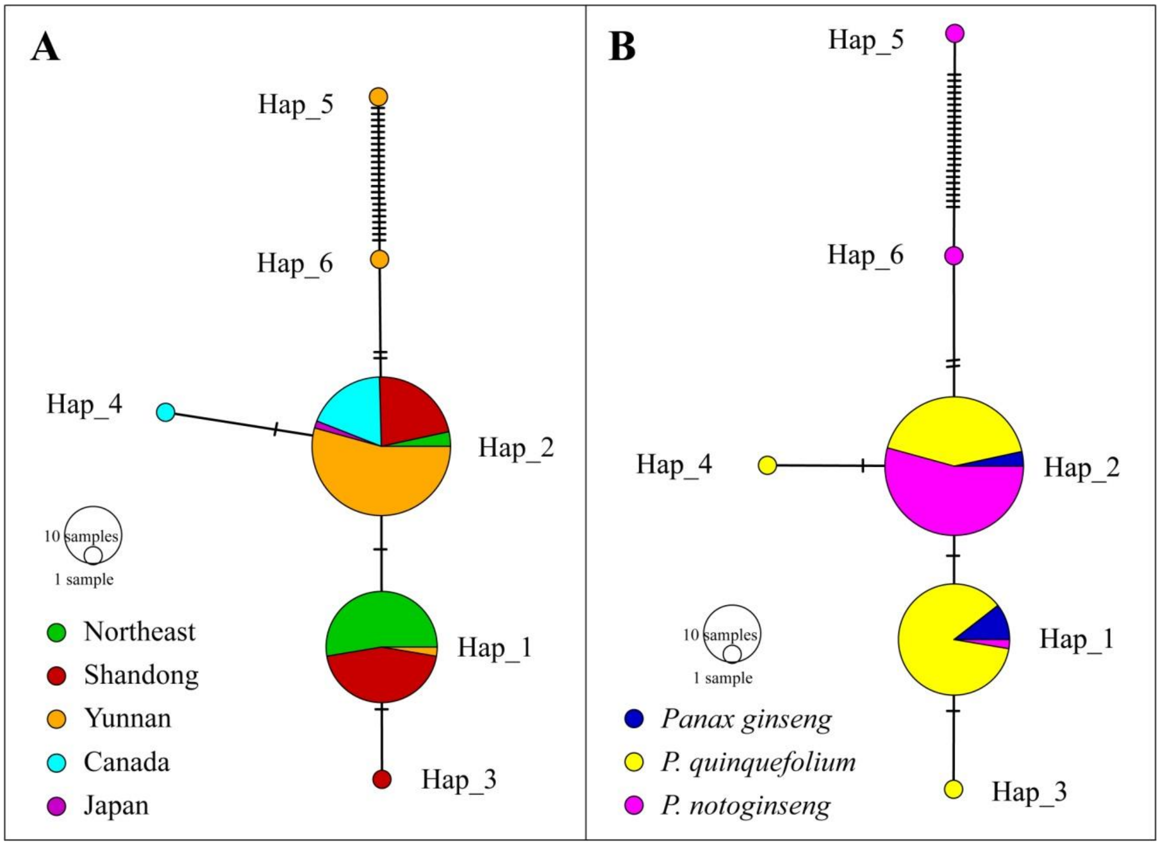

Figure S8. TCS haplotype network generated based on partial sequence of the *his3* gene representing worldwide *Ilyonectria mors-panacis* isolates. A: geographical populations; B: populations divided based on hosts. The size of the circle represents the haplotype frequencies in populations. Hatch marks indicate the number of mutations.

Table S1. CLF isolates obtained from *Panax ginseng* or *P. quinquefolius* in Northeast China

Isolate number	Species	Host	Location(province, city, county, town)	Year collected	Haplotype	GenBank accession number	Population code	Latitude, longitude
301	*I. communis*	*P. ginseng*	Jilin, Baishan, Changbai, Baoquanshan	2012	Hap_1	MF350433	JBC	41.52N, 127.46E
320	*I. changbaiensis*	*P. ginseng*	Jilin, Baishan, Changbai, Baoquanshan	2012	Hap_2	MF350443	JBC	41.52N, 127.46E
408	*I. communis*	*P. ginseng*	Jilin, Baishan, Changbai, Baoquanshan	2012	Hap_1	-	JBC	41.52N, 127.46E
423	*I. communis*	*P. ginseng*	Jilin, Baishan, Changbai, Baoquanshan	2012	Hap_1	-	JBC	41.52N, 127.46E
608	*I. communis*	*P. ginseng*	Jilin, Baishan, Changbai, Baoquanshan	2012	Hap_1	-	JBC	41.52N, 127.46E
610	*I. changbaiensis*	*P. ginseng*	Jilin, Baishan, Changbai, Baoquanshan	2012	Hap_5	MT043275	JBC	41.52N, 127.46E
616	*I. communis*	*P. ginseng*	Jilin, Baishan, Changbai, Baoquanshan	2012	Hap_1	-	JBC	41.52N, 127.46E
1506	*I. changbaiensis*	*P. ginseng*	Jilin, Baishan, Changbai, Baoquanshan	2012	Hap_2	MF350440	JBC	41.52N, 127.46E
1512	*I. communis*	*P. ginseng*	Jilin, Baishan, Changbai, Baoquanshan	2012	Hap_1	-	JBC	41.52N, 127.46E
1524	*I. communis*	*P. ginseng*	Jilin, Baishan, Changbai, Baoquanshan	2012	Hap_1	-	JBC	41.52N, 127.46E
1606	*I. communis*	*P. ginseng*	Jilin, Baishan, Changbai, Baoquanshan	2012	Hap_1	-	JBC	41.52N, 127.46E
1712	*I. communis*	*P. ginseng*	Jilin, Baishan, Changbai, Baoquanshan	2012	Hap_1	-	JBC	41.52N, 127.46E
1803	*I. changbaiensis*	*P. ginseng*	Jilin, Baishan, Changbai, Baoquanshan	2012	Hap_2	MF350441	JBC	41.52N, 127.46E
4404	*I. changbaiensis*	*P. ginseng*	Jilin, Baishan, Changbai, Baoquanshan	2012	Hap_2	MF350437	JBC	41.52N, 127.46E
YH12	*I. robusta*	*P. quinquefolius*	Heilongjiang, Harbin, Yilan, Yilan	2014	Hap_8	-	HHYT	46.32N, 129.56E
YH15	*I. robusta*	*P. quinquefolius*	Heilongjiang, Harbin, Yilan, Yilan	2014	Hap_8	-	HHYT	46.32N, 129.56E
YH16	*I. robusta*	*P. quinquefolius*	Heilongjiang, Harbin, Yilan, Yilan	2014	Hap_8	-	HHYT	46.32N, 129.56E
YH17	*I. robusta*	*P. quinquefolius*	Heilongjiang, Harbin, Yilan, Yilan	2014	Hap_8	-	HHYT	46.32N, 129.56E
YH18	*I. robusta*	*P. quinquefolius*	Heilongjiang, Harbin, Yilan, Yilan	2014	Hap_8	-	HHYT	46.32N, 129.56E
YH19	*I. robusta*	*P. quinquefolius*	Heilongjiang, Harbin, Yilan, Yilan	2014	Hap_8	-	HHYT	46.32N, 129.56E
YJ11	*I. mors-panacis*	*P. quinquefolius*	Jilin, Baishan, Fusong Beigang	2014	Hap_3	-	JBF	42.42N, 127.54E
YJ12	*I. robusta*	*P. quinquefolius*	Jilin, Baishan, Fusong Beigang	2014	Hap_17	-	JBF	42.42N, 127.54E
YJ17	*I. mors-panacis*	*P. quinquefolius*	Jilin, Baishan, Fusong Beigang	2014	Hap_18	MT043277	JBF	42.42N, 127.54E
YJ19	*I. mors-panacis*	*P. quinquefolius*	Jilin, Baishan, Fusong Beigang	2014	Hap_3	-	JBF	42.42N, 127.54E
YJ23	*I. robusta*	*P. quinquefolius*	Jilin, Baishan, Fusong, Lushuihe	2014	Hap_19	MT043274	JBF	42.42N, 127.54E
YJ25	*I. robusta*	*P. quinquefolius*	Jilin, Baishan, Fusong, Lushuihe	2014	Hap_17	-	JBF	42.42N, 127.54E
YJ26	*I. robusta*	*P. quinquefolius*	Jilin, Baishan, Fusong, Lushuihe	2014	Hap_9	-	JBF	42.42N, 127.54E
YJ28	*I. communis*	*P. quinquefolius*	Jilin, Baishan, Fusong, Lushuihe	2014	Hap_1	-	JBF	42.42N, 127.54E
YJ29	*I. communis*	*P. quinquefolius*	Jilin, Baishan, Fusong, Lushuihe	2014	Hap_1	-	JBF	42.42N, 127.54E
YJ52	*I. communis*	*P. quinquefolius*	Jilin, Jilin, Jiaohe, Qianjin	2014	Hap_6	-	JJJ	43.63N, 127.73E
YJ54	*I. robusta*	*P. quinquefolius*	Jilin, Jilin, Jiaohe, Qianjin	2014	Hap_11	-	JJJ	43.63N, 127.73E
YJ56	*I. robusta*	*P. quinquefolius*	Jilin, Jilin, Jiaohe, Qianjin	2014	Hap_9	-	JJJ	43.63N, 127.73E
YJ57	*I. communis*	*P. quinquefolius*	Jilin, Jilin, Jiaohe, Qianjin	2014	Hap_1	-	JJJ	43.63N, 127.73E

YJ58	*I. communis*	*P. quinquefolius*	Jilin, Jilin, Jiaohe, Qianjin	2014	Hap_1	-	JJJ	43.63N, 127.73E
YJ59	*I. robusta*	*P. quinquefolius*	Jilin, Jilin, Jiaohe, Qianjin	2014	Hap_9	-	JJJ	43.63N, 127.73E
YJ71	*I. robusta*	*P. quinquefolius*	Jilin, Yanbian, Wangqing, Daxinggou	2014	Hap_8	-	JYW	43.42N, 129.66E
YJ76	*I. robusta*	*P. quinquefolius*	Jilin, Yanbian, Wangqing, Daxinggou	2014	Hap_9	-	JYW	43.42N, 129.66E
YJ78	*I. robusta*	*P. quinquefolius*	Jilin, Yanbian, Wangqing, Daxinggou	2014	Hap_9	-	JYW	43.42N, 129.66E
YJ79	*I. robusta*	*P. quinquefolius*	Jilin, Yanbian, Wangqing, Daxinggou	2014	Hap_9	-	JYW	43.42N, 129.66E
YJ83	*I. mors-panacis*	*P. quinquefolius*	Jilin, Yanbian, Hunchun, Yangpao	2014	Hap_3	-	JYH	42.90N, 130.50E
11R1	*I. communis*	*P. ginseng*	Jilin, Baishan, Changbai, Baoquanshan	2012	Hap_1	-	JBC	41.52N, 127.46E
11R7	*I. communis*	*P. ginseng*	Jilin, Baishan, Changbai, Baoquanshan	2012	Hap_1	-	JBC	41.52N, 127.46E
11R8-2	*I. changbaiensis*	*P. ginseng*	Jilin, Baishan, Changbai, Baoquanshan	2012	Hap_2	MF350439	JBC	41.52N, 127.46E
11R9	*I. mors-panacis*	*P. ginseng*	Jilin, Baishan, Changbai, Baoquanshan	2012	Hap_3	MF350450	JBC	41.52N, 127.46E
308-2	*I. communis*	*P. ginseng*	Jilin, Baishan, Changbai, Baoquanshan	2012	Hap_1	-	JBC	41.52N, 127.46E
3S04	*I. communis*	*P. ginseng*	Jilin, Baishan, Changbai, Baoquanshan	2012	Hap_1	-	JBC	41.52N, 127.46E
3S07	*D. hordeicola*	*P. ginseng*	Jilin, Baishan, Changbai, Baoquanshan	2012	Hap_4	MF350455	JBC	41.52N, 127.46E
3S12	*I. communis*	*P. ginseng*	Jilin, Baishan, Changbai, Baoquanshan	2012	Hap_1	-	JBC	41.52N, 127.46E
3S24	*I. communis*	*P. ginseng*	Jilin, Baishan, Changbai, Baoquanshan	2012	Hap_1	-	JBC	41.52N, 127.46E
3S26	*I. communis*	*P. ginseng*	Jilin, Baishan, Changbai, Baoquanshan	2012	Hap_1	-	JBC	41.52N, 127.46E
71R2	*I. communis*	*P. ginseng*	Jilin, Baishan, Changbai, Baoquanshan	2012	Hap_6	MF350431	JBC	41.52N, 127.46E
71R7	*I. robusta*	*P. ginseng*	Jilin, Baishan, Changbai, Baoquanshan	2012	Hap_7	MT043271	JBC	41.52N, 127.46E
73P1	*I. communis*	*P. ginseng*	Jilin, Baishan, Changbai, Baoquanshan	2012	Hap_1	-	JBC	41.52N, 127.46E
73R4	*I. communis*	*P. ginseng*	Jilin, Baishan, Changbai, Baoquanshan	2012	Hap_1	-	JBC	41.52N, 127.46E
74R2	*I. communis*	*P. ginseng*	Jilin, Baishan, Changbai, Baoquanshan	2012	Hap_1	-	JBC	41.52N, 127.46E
H110	*I. robusta*	*P. ginseng*	Heilongjiang, Harbin, Tonghe, Fengshar	2013	Hap_7	-	HHYT	45.75N, 131.19E
H114	*I. robusta*	*P. ginseng*	Heilongjiang, Harbin, Tonghe, Fengshar	2013	Hap_8	-	HHYT	45.75N, 131.19E
H201	*I. robusta*	*P. ginseng*	Heilongjiang, Heihe, Beian, Tongbei	2013	Hap_9	-	HHB	47.77N, 126.78E
H2022	*I. robusta*	*P. ginseng*	Heilongjiang, Heihe, Beian, Tongbei	2013	Hap_9	-	HHB	47.77N, 126.78E
H203	*I. robusta*	*P. ginseng*	Heilongjiang, Heihe, Beian, Tongbei	2013	Hap_9	-	HHB	47.77N, 126.78E
H2062	*I. robusta*	*P. ginseng*	Heilongjiang, Heihe, Beian, Tongbei	2013	Hap_9	-	HHB	47.77N, 126.78E
H207	*I. robusta*	*P. ginseng*	Heilongjiang, Heihe, Beian, Tongbei	2013	Hap_9	MF350434	HHB	47.77N, 126.78E
H2073	*I. communis*	*P. ginseng*	Heilongjiang, Heihe, Beian, Tongbei	2013	Hap_1	MF350434	HHB	47.77N, 126.78E
H209	*I. robusta*	*P. ginseng*	Heilongjiang, Heihe, Beian, Tongbei	2013	Hap_9	-	HHB	47.77N, 126.78E
H301	*I. robusta*	*P. ginseng*	Heilongjiang, Qitaihe, Qiezihe, Tieshan	2013	Hap_9	-	HQQ	45.75N, 131.19E
H302	*I. robusta*	*P. ginseng*	Heilongjiang, Qitaihe, Qiezihe, Tieshan	2013	Hap_9	-	HQQ	45.75N, 131.19E
H310	*I. robusta*	*P. ginseng*	Heilongjiang, Qitaihe, Qiezihe, Tieshan	2013	Hap_11	-	HQQ	45.75N, 131.19E
J101	*I. communis*	*P. ginseng*	Jilin, Baishan, Fusong, Beigang	2013	Hap_1	MF350432	JBF	42.42N, 127.54E

J103	*I. communis*	*P. ginseng*	Jilin, Baishan, Fusong, Beigang	2013	Hap_1	-	JBF	42.42N, 127.54E
J104	*I. communis*	*P. ginseng*	Jilin, Baishan, Fusong, Beigang	2013	Hap_12	MT043276	JBF	42.42N, 127.54E
J105	*I. robusta*	*P. ginseng*	Jilin, Baishan, Fusong, Beigang	2013	Hap_9	-	JBF	42.42N, 127.54E
J107	*I. robusta*	*P. ginseng*	Jilin, Baishan, Fusong, Beigang	2013	Hap_9	-	JBF	42.42N, 127.54E
J1073	*I. communis*	*P. ginseng*	Jilin, Baishan, Fusong, Beigang	2013	Hap_1	-	JBF	42.42N, 127.54E
J108	*I. communis*	*P. ginseng*	Jilin, Baishan, Fusong, Beigang	2013	Hap_1	-	JBF	42.42N, 127.54E
J108-2	*I. robusta*	*P. ginseng*	Jilin, Baishan, Fusong, Beigang	2013	Hap_9	-	JBF	42.42N, 127.54E
J109	*I. communis*	*P. ginseng*	Jilin, Baishan, Fusong, Beigang	2013	Hap_1	-	JBF	42.42N, 127.54E
J2072	*I. communis*	*P. ginseng*	Jilin, Baishan, Fusong, Xigang	2013	Hap_1	-	JBF	42.42N, 127.54E
J2102	*I. communis*	*P. ginseng*	Jilin, Baishan, Fusong, Xigang	2013	Hap_1	-	JBF	42.42N, 127.54E
J214	*I. communis*	*P. ginseng*	Jilin, Baishan, Fusong, Xigang	2013	Hap_1	-	JBF	42.42N, 127.54E
J302	*I. communis*	*P. ginseng*	Jilin, Baishan, Changbai, Malugou	2013	Hap_1	-	JBCM	41.44N, 128.21E
J3032	*I. robusta*	*P. ginseng*	Jilin, Baishan, Changbai, Malugou	2013	Hap_9	-	JBCM	41.44N, 128.21E
J304	*I. robusta*	*P. ginseng*	Jilin, Baishan, Changbai, Malugou	2013	Hap_9	-	JBCM	41.44N, 128.21E
J305	*I. communis*	*P. ginseng*	Jilin, Baishan, Changbai, Malugou	2013	Hap_1	-	JBCM	41.44N, 128.21E
J3093	*I. communis*	*P. ginseng*	Jilin, Baishan, Changbai, Malugou	2013	Hap_1	-	JBCM	41.44N, 128.21E
J3102	*I. communis*	*P. ginseng*	Jilin, Baishan, Changbai, Malugou	2013	Hap_1	-	JBCM	41.44N, 128.21E
J322	*Ilyonectria* sp.	*Panax ginseng*	Jilin, Baishan, Changbai, Malugou	2013	Hap_13	MT043270	JBCM	41.44N, 128.21E
J410	*I. communis*	*P. ginseng*	Jilin, Baishan, Changbai, Shisidaogou	2013	Hap_1	MF350430	JBC	41.52N, 127.46E
J421	*I. communis*	*P. ginseng*	Jilin, Baishan, Changbai, Shisidaogou	2013	Hap_1	-	JBC	41.52N, 127.46E
J5032	*I. robusta*	*P. ginseng*	Jilin, Jilin, Jiaohe, Huangsongdian	2013	Hap_9	-	JJJ	43.63N, 127.73E
J506	*I. robusta*	*P. ginseng*	Jilin, Jilin, Jiaohe, Huangsongdian	2013	Hap_9	-	JJJ	43.63N, 127.73E
H309	*I. qitaiheensis*	*P. ginseng*	Heilongjiang, Qitaihe, Qiezihe, Tieshan	2013	Hap_10	MF350445	HQQ	45.75N, 131.19E
J9191	*I. qitaiheensis*	*P. ginseng*	Jilin, Tonghua, Jian, Toudao	2013	Hap_16	MF350446	JTJ	41.51N, 125.88E
J601	*I. communis*	*P. ginseng*	Jilin, Yanbian, Antu, Songjiang	2013	Hap_1	-	JYAD	42.58N, 128.33E
J606	*I. robusta*	*P. ginseng*	Jilin, Yanbian, Antu, Songjiang	2013	Hap_11	-	JYAD	42.58N, 128.33E
J608	*I. robusta*	*P. ginseng*	Jilin, Yanbian, Antu, Songjiang	2013	Hap_9	-	JYAD	42.58N, 128.33E
J610	*I. robusta*	*P. ginseng*	Jilin, Yanbian, Antu, Songjiang	2013	Hap_9	-	JYAD	42.58N, 128.33E
J701	*I. communis*	*P. ginseng*	Jilin, Yanbian, Antu, Xinhexiang	2013	Hap_1	-	JYAD	42.58N, 128.33E
J702	*I. robusta*	*P. ginseng*	Jilin, Yanbian, Antu, Xinhexiang	2013	Hap_11	-	JYAD	42.58N, 128.33E
J703	*I. robusta*	*P. ginseng*	Jilin, Yanbian, Antu, Xinhexiang	2013	Hap_11	-	JYAD	42.58N, 128.33E
J706	*I. robusta*	*P. ginseng*	Jilin, Yanbian, Antu, Xinhexiang	2013	Hap_9	-	JYAD	42.58N, 128.33E
J709	*I. communis*	*P. ginseng*	Jilin, Yanbian, Antu, Xinhexiang	2013	Hap_1	-	JYAD	42.58N, 128.33E
J710	*I. communis*	*P. ginseng*	Jilin, Yanbian, Antu, Xinhexiang	2013	Hap_1	MF350435	JYAD	42.58N, 128.33E
J711	*Dactylonectria* sp.	*P. ginseng*	Jilin, Yanbian, Antu, Xinhexiang	2013	Hap_14	MF350452	JYAD	42.58N, 128.33E

J713	*I. robusta*	*P. ginseng*	Jilin, Yanbian, Antu, Xinhexiang	2013	Hap_8	-	JYAD	42.58N, 128.33E
J801	*I. robusta*	*P. ginseng*	Jilin, Yanbian, Hunchun, Hadamen	2013	Hap_8	-	JYH	42.93N, 130.49E
J802	*I. robusta*	*P. ginseng*	Jilin, Yanbian, Hunchun, Hadamen	2013	Hap_9	-	JYH	42.93N, 130.49E
J803	*I. robusta*	*P. ginseng*	Jilin, Yanbian, Hunchun, Hadamen	2013	Hap_9	-	JYH	42.93N, 130.49E
J804	*I. robusta*	*P. ginseng*	Jilin, Yanbian, Hunchun, Hadamen	2013	Hap_11	-	JYH	42.93N, 130.49E
J811	*I. robusta*	*P. ginseng*	Jilin, Yanbian, Hunchun, Hadamen	2013	Hap_11	-	JYH	42.93N, 130.49E
J812	*I. robusta*	*P. ginseng*	Jilin, Yanbian, Hunchun, Hadamen	2013	Hap_9	-	JYH	42.93N, 130.49E
J901	*I. robusta*	*P. ginseng*	Jilin, Tonghua, Jian, Toudao	2013	Hap_9	-	JTJ	41.51N, 125.88E
J903	*I. robusta*	*P. ginseng*	Jilin, Tonghua, Jian, Toudao	2013	Hap_9	-	JTJ	41.51N, 125.88E
J904	*I. robusta*	*P. ginseng*	Jilin, Tonghua, Jian, Toudao	2013	Hap_9	-	JTJ	41.51N, 125.88E
J905	*I. robusta*	*P. ginseng*	Jilin, Tonghua, Jian, Toudao	2013	Hap_9	-	JTJ	41.51N, 125.88E
J906	*I. robusta*	*P. ginseng*	Jilin, Tonghua, Jian, Toudao	2013	Hap_9	KM015299	JTJ	41.51N, 125.88E
J907	*I. robusta*	*P. ginseng*	Jilin, Tonghua, Jian, Toudao	2013	Hap_9	-	JTJ	41.51N, 125.88E
J909	*I. robusta*	*P. ginseng*	Jilin, Tonghua, Jian, Toudao	2013	Hap_9	-	JTJ	41.51N, 125.88E
J913	*I. robusta*	*P. ginseng*	Jilin, Tonghua, Jian, Toudao	2013	Hap_7	-	JTJ	41.51N, 125.88E
J914	*I. robusta*	*P. ginseng*	Jilin, Tonghua, Jian, Toudao	2013	Hap_9	-	JTJ	41.51N, 125.88E
J915	*I. robusta*	*P. ginseng*	Jilin, Tonghua, Jian, Toudao	2013	Hap_9	-	JTJ	41.51N, 125.88E
J917	*I. robusta*	*P. ginseng*	Jilin, Tonghua, Jian, Toudao	2013	Hap_15	MT043272	JTJ	41.51N, 125.88E
J918	*I. robusta*	*P. ginseng*	Jilin, Tonghua, Jian, Toudao	2013	Hap_9	-	JTJ	41.51N, 125.88E
J91933	*I. robusta*	*P. ginseng*	Jilin, Tonghua, Jian, Toudao	2013	Hap_9	-	JTJ	41.51N, 125.88E
J922	*I. robusta*	*P. ginseng*	Jilin, Tonghua, Jian, Toudao	2013	Hap_9	-	JTJ	41.51N, 125.88E
J923	*I. robusta*	*P. ginseng*	Jilin, Tonghua, Jian, Toudao	2013	Hap_9	-	JTJ	41.51N, 125.88E
YH11	*I. robusta*	*P. quinquefolius*	Heilongjiang, Harbin, Yilan, Yilan	2014	Hap_8	-	HHYT	46.32N, 129.56E
YH1101	*I. robusta*	*P. quinquefolius*	Heilongjiang, Harbin, Yilan, Yilan	2014	Hap_8	-	HHYT	46.32N, 129.56E
YH1102	*I. communis*	*P. quinquefolius*	Heilongjiang, Harbin, Yilan, Yilan	2014	Hap_1	-	HHYT	46.32N, 129.56E
YH111	*I. communis*	*P. quinquefolius*	Heilongjiang, Harbin, Yilan, Yilan	2014	Hap_6	-	HHYT	46.32N, 129.56E
YH112	*I. robusta*	*P. quinquefolius*	Heilongjiang, Harbin, Yilan, Yilan	2014	Hap_8	-	HHYT	46.32N, 129.56E
YH113	*I. robusta*	*P. quinquefolius*	Heilongjiang, Harbin, Yilan, Yilan	2014	Hap_8	-	HHYT	46.32N, 129.56E
YH114	*I. robusta*	*P. quinquefolius*	Heilongjiang, Harbin, Yilan, Yilan	2014	Hap_8	-	HHYT	46.32N, 129.56E
YJ110	*Dactylonectria* sp.	*P. quinquefolius*	Jilin, Baishan, Fusong Beigang	2014	Hap_14	-	JBF	42.42N, 127.54E
YJ112	*I. mors-panacis*	*P. quinquefolius*	Jilin, Baishan, Fusong Beigang	2014	Hap_3	-	JBF	42.42N, 127.54E
YJ115	*I. mors-panacis*	*P. quinquefolius*	Jilin, Baishan, Fusong Beigang	2014	Hap_3	-	JBF	42.42N, 127.54E
YJ117	*I. mors-panacis*	*P. quinquefolius*	Jilin, Baishan, Fusong Beigang	2014	Hap_3	-	JBF	42.42N, 127.54E
YJ210	*I. robusta*	*P. quinquefolius*	Jilin, Baishan, Fusong, Lushuihe	2014	Hap_9	-	JBF	42.42N, 127.54E
YJ211	*I. communis*	*P. quinquefolius*	Jilin, Baishan, Fusong, Lushuihe	2014	Hap_1	-	JBF	42.42N, 127.54E

YJ212	*Dactylonectria* sp.	*P. quinquefolius*	Jilin, Baishan, Fusong, Lushuihe	2014	Hap_14	-	JBF	42.42N, 127.54E
YJ310	*I. communis*	*P. quinquefolius*	Jilin, Baishan, Changbai, Malugou	2014	Hap_1	-	JBCM	41.44N, 128.21E
YJ312	*I. communis*	*P. quinquefolius*	Jilin, Baishan, Changbai, Malugou	2014	Hap_1	-	JBCM	41.44N, 128.21E
YJ315	*I. communis*	*P. quinquefolius*	Jilin, Baishan, Changbai, Malugou	2014	Hap_1	-	JBCM	41.44N, 128.21E
YJ322	*I. robusta*	*P. quinquefolius*	Jilin, Baishan, Changbai, Malugou	2014	Hap_11	-	JBCM	41.44N, 128.21E
YJ327	*I. mors-panacis*	*P. quinquefolius*	Jilin, Baishan, Changbai, Malugou	2014	Hap_3	-	JBCM	41.44N, 128.21E
YJ3281	*I. mors-panacis*	*P. quinquefolius*	Jilin, Baishan, Changbai, Malugou	2014	Hap_3	-	JBCM	41.44N, 128.21E
YJ410	*I. communis*	*P. quinquefolius*	Jilin, Baishan, Linjiang, Dongbeicha	2014	Hap_1	-	JBL	41.74N, 127.29E
YJ4113	*I. communis*	*P. quinquefolius*	Jilin, Baishan, Linjiang, Dongbeicha	2014	Hap_1	-	JBL	41.74N, 127.29E
YJ4114	*I. communis*	*P. quinquefolius*	Jilin, Baishan, Linjiang, Dongbeicha	2014	Hap_1	-	JBL	41.74N, 127.29E
YJ4116	*I. communis*	*P. quinquefolius*	Jilin, Baishan, Linjiang, Dongbeicha	2014	Hap_1	-	JBL	41.74N, 127.29E
YJ4117	*I. communis*	*P. quinquefolius*	Jilin, Baishan, Linjiang, Dongbeicha	2014	Hap_1	-	JBL	41.74N, 127.29E
YJ4118	*I. communis*	*P. quinquefolius*	Jilin, Baishan, Linjiang, Dongbeicha	2014	Hap_1	-	JBL	41.74N, 127.29E
YJ413	*I. mors-panacis*	*P. quinquefolius*	Jilin, Baishan, Linjiang, Dongbeicha	2014	Hap_3	-	JBL	41.74N, 127.29E
YJ415	*I. communis*	*P. quinquefolius*	Jilin, Baishan, Linjiang, Dongbeicha	2014	Hap_1	-	JBL	41.74N, 127.29E
YJ417	*I. communis*	*P. quinquefolius*	Jilin, Baishan, Linjiang, Dongbeicha	2014	Hap_1	-	JBL	41.74N, 127.29E
YJ418	*I. communis*	*P. quinquefolius*	Jilin, Baishan, Linjiang, Dongbeicha	2014	Hap_1	-	JBL	41.74N, 127.29E
YJ419	*I. communis*	*P. quinquefolius*	Jilin, Baishan, Linjiang, Dongbeicha	2014	Hap_1	-	JBL	41.74N, 127.29E
YJ423	*I. mors-panacis*	*P. quinquefolius*	Jilin, Baishan, Linjiang, Dongbeicha	2014	Hap_3	-	JBL	41.74N, 127.29E
YJ424	*I. mors-panacis*	*P. quinquefolius*	Jilin, Baishan, Linjiang, Dongbeicha	2014	Hap_3	-	JBL	41.74N, 127.29E
YJ4251	*I. mors-panacis*	*P. quinquefolius*	Jilin, Baishan, Linjiang, Dongbeicha	2014	Hap_3	-	JBL	41.74N, 127.29E
YJ4252	*I. mors-panacis*	*P. quinquefolius*	Jilin, Baishan, Linjiang, Dongbeicha	2014	Hap_3	-	JBL	41.74N, 127.29E
YJ510	*I. robusta*	*P. quinquefolius*	Jilin, Jilin, Jiaohe, Qianjin	2014	Hap_20	MT043273	JJJ	43.63N, 127.73E
YJ511	*I. mors-panacis*	*P. quinquefolius*	Jilin, Jilin, Jiaohe, Qianjin	2014	Hap_3	-	JJJ	43.63N, 127.73E
YJ513	*I. robusta*	*P. quinquefolius*	Jilin, Jilin, Jiaohe, Qianjin	2014	Hap_11	-	JJJ	43.63N, 127.73E
YJ5151	*I. robusta*	*P. quinquefolius*	Jilin, Jilin, Jiaohe, Qianjin	2014	Hap_9	-	JJJ	43.63N, 127.73E
YJ5152	*Dactylonectria* sp.	*P. quinquefolius*	Jilin, Jilin, Jiaohe, Qianjin	2014	Hap_14	-	JJJ	43.63N, 127.73E
YJ612	*I. mors-panacis*	*P. quinquefolius*	Jilin, Yanbian, Dunhua, Dapuchaihe	2014	Hap_3	-	JYAD	42.88N, 128.03E
YJ6171	*I. mors-panacis*	*P. quinquefolius*	Jilin, Yanbian, Dunhua, Dapuchaihe	2014	Hap_3	-	JYAD	42.88N, 128.03E
YJ710	*I. robusta*	*P. quinquefolius*	Jilin, Yanbian, Wangqing, Daxinggou	2014	Hap_8	-	JYW	43.42N, 129.66E
YJ711	*I. robusta*	*P. quinquefolius*	Jilin, Yanbian, Wangqing, Daxinggou	2014	Hap_7	-	JYW	43.42N, 129.66E

"-" means that the isolate shares the same sequences with other isolates belonging to the same haplotype.Therefore at least one GenBank accession number was provided for each haplotype.

Table S2. CLF isolates used in worldwide genetic diversity and population structure analyses

Isolate number	Species	Host	Location	GenBank accession number	Population code
CBS 321.34	*Ilyonectria robusta*	*Loroglossum hircinum*	Tunis, Tunisia	JF735517	Africa
CBS 308.35	*I. robusta*	*Panax quinquefolium*	Ontario, Canada	JF735518	Ameirica/Canada
CBS 773.83	*I. robusta*	Water, in aquarium with Anodonta	Utrecht, Netherlands	JF735519	Europe
CBS 605.92	*I. robusta*	*Tilia petiolaris*	Hamburg, Germany	JF735520	Europe
CBS 117814	*I. robusta*	*Quercus* sp.	Patzmannsdorf, Austria	JF735521	Oceania
CBS 117815	*I. robusta*	*Quercus* sp.	Patzmannsdorf, Austria	JF735522	Oceania
CBS 117818	*I. robusta*	*Quercus* sp.	Patzmannsdorf, Austria	JF735523	Oceania
CBS 117820	*I. robusta*	*Quercus robur*	Niederweiden, Austria	JF735524	Oceania
CBS 117821	*I. robusta*	*Quercus robur*	Niederweiden, Austria	JF735525	Oceania
CBS 117822	*I. robusta*	*Quercus robur*	Niederweiden, Austria	JF735526	Oceania
CBS 117823	*I. robusta*	*Quercus robur*	Niederweiden, Austria	JF735527	Oceania
CD1666	*I. robusta*	*Panax quinquefolium*	Nova Scotia, Canada	JF735528	Ameirica/Canada
CPC 13532	*I. robusta*	*Prunus cerasus*	Ontario, Canada	JF735529	Ameirica
Cy23	*I. robusta*	*Vitis* sp.	Ribatejo e Oeste, Portugal	JF735530	Europe
Cy158	*I. robusta*	*Vitis vinifera*	Lamego, Cambres, Portugal	JF735531	Europe
CBS 129084	*I. robusta*	*Vitis vinifera*	Mon ção, Portugal	JF735532	Europe
Cy231	*I. robusta*	*Thymus* sp.	Lisbon, Portugal	JF735533	Europe
10-K-TR-3	*I. robusta*	*Actinidia chinensis*	Turkey	JQ860004	Europe
TRR-2	*I. robusta*	*Actinidia chinensis*	Turkey	JQ860005	Europe
Cyl 16	*I. robusta*	*Eriobotrya japonica*	Spain	KC514078	Europe
Cy9UFSM	*I. robusta*	*Vitis vinifera*	Southern Brazil	KF633172	Ameirica
ACK-10271	*I. robusta*	*Aconitum kongboense*	Tibet, China	KJ710516	Asia
ACK-10272	*I. robusta*	*Aconitum kongboense*	Tibet, China	KJ710517	Asia
Cyl131	*I. robusta*	*Malus pumila*	South Tyrol, Italy	KP823902	Europe
Cy-FO-217	*I. robusta*	Forest nurseries	Spain	KX709582	Europe
EFA 184	*I. robusta*	*Vitis vinifera*	unknown/Spain	MF471467	Europe
UFAH00042	*I. robusta*	*Rubus glaucus*	Ecuador	MG852007	Ameirica
BV-816	*I. robusta*	*Vitis vinifera*	Spain	MH229866	Europe
GIHF-156	*I. robusta*	*Vitis vinifera*	Spain	MH229867	Europe
ICMP14133	*I. robusta*	*Vitis vinifera*	unknown/New Zealand	MH553544	Oceania

8916	*I. robusta*	Stone fruit	South Africa	MK765797	Africa
JBLLJ-3	*I. robusta*	*P. ginseng*	Northeast, China	MN101765	Asia/Northeast
JYDDH-1	*I. robusta*	*P. ginseng*	Northeast, China	MN101766	Asia/Northeast
JYAED-2	*I. robusta*	*P. ginseng*	Northeast, China	MN101767	Asia/Northeast
JJHHD-6	*I. robusta*	*P. ginseng*	Northeast, China	MN101768	Asia/Northeast
HSSSL-8	*I. robusta*	*P. ginseng*	Northeast, China	MN101769	Asia/Northeast
HYTTL-4	*I. robusta*	*P. ginseng*	Northeast, China	MN101770	Asia/Northeast
JYHCH-1	*I. robusta*	*P. ginseng*	Northeast, China	MN101771	Asia/Northeast
JBCML-3	*I. robusta*	*P. ginseng*	Northeast, China	MN101772	Asia/Northeast
JTJYL-11	*I. robusta*	*P. ginseng*	Northeast, China	MN101773	Asia/Northeast
CBS 120359	*I. mors-panacis*	*P. quinquefolius*	Ontario, Canada	JF735547	Ameirica/Canada
CBS 120361	*I. mors-panacis*	*P. quinquefolius*	Ontario, Canada	JF735548	Ameirica/Canada
CBS 120364	*I. mors-panacis*	*P. quinquefolius*	Ontario, Canada	JF735549	Ameirica/Canada
CBS 120365	*I. mors-panacis*	*P. quinquefolius*	Ontario, Canada	JF735550	Ameirica/Canada
CBS 120366	*I. mors-panacis*	*P. quinquefolius*	Ontario, Canada	JF735551	Ameirica/Canada
CBS 120367	*I. mors-panacis*	*P. quinquefolius*	Ontario, Canada	JF735552	Ameirica/Canada
CBS 120368	*I. mors-panacis*	*P. quinquefolius*	Ontario, Canada	JF735553	Ameirica/Canada
CBS 120369	*I. mors-panacis*	*P. quinquefolius*	Ontario, Canada	JF735554	Ameirica/Canada
CPC 13535	*I. mors-panacis*	*P. quinquefolius*	Ontario, Canada	JF735555	Ameirica/Canada
CPC 13537	*I. mors-panacis*	*P. quinquefolius*	Ontario, Canada	JF735556	Ameirica/Canada
CBS 306.35	*I. mors-panacis*	*P. quinquefolius*	Ontario, Canada	JF735557	Ameirica/Canada
CBS 307.35	*I. mors-panacis*	*P. quinquefolius*	Ontario, Canada	JF735558	Ameirica/Canada
CBS 124662	*I. mors-panacis*	*P. ginseng*	Nagano, Japan	JF735559	Japan
FG003	*I. mors-panacis*	*P. notoginseng*	Yunnan, China	MK270539	Yunnan
FG004	*I. mors-panacis*	*P. notoginseng*	Yunnan, China	MK270540	Yunnan
FG009	*I. mors-panacis*	*P. notoginseng*	Yunnan, China	MK270545	Yunnan
FG010	*I. mors-panacis*	*P. notoginseng*	Yunnan, China	MK270546	Yunnan
FG011	*I. mors-panacis*	*P. notoginseng*	Yunnan, China	MK270547	Yunnan
FG012	*I. mors-panacis*	*P. notoginseng*	Yunnan, China	MK270548	Yunnan
FG013	*I. mors-panacis*	*P. notoginseng*	Yunnan, China	MK270549	Yunnan
FG014	*I. mors-panacis*	*P. notoginseng*	Yunnan, China	MK270550	Yunnan
FG015	*I. mors-panacis*	*P. notoginseng*	Yunnan, China	MK270551	Yunnan

FG016	*I. mors-panacis*	*P. notoginseng*	Yunnan, China	MK270552	Yunnan
FG018	*I. mors-panacis*	*P. notoginseng*	Yunnan, China	MK270554	Yunnan
FG023	*I. mors-panacis*	*P. notoginseng*	Yunnan, China	MK270559	Yunnan
FG024	*I. mors-panacis*	*P. notoginseng*	Yunnan, China	MK270560	Yunnan
FG025	*I. mors-panacis*	*P. notoginseng*	Yunnan, China	MK270561	Yunnan
FG027	*I. mors-panacis*	*P. notoginseng*	Yunnan, China	MK270563	Yunnan
FG028	*I. mors-panacis*	*P. notoginseng*	Yunnan, China	MK270564	Yunnan
FG030	*I. mors-panacis*	*P. notoginseng*	Yunnan, China	MK270566	Yunnan
FG031	*I. mors-panacis*	*P. notoginseng*	Yunnan, China	MK270567	Yunnan
FG032	*I. mors-panacis*	*P. notoginseng*	Yunnan, China	MK270568	Yunnan
FG033	*I. mors-panacis*	*P. notoginseng*	Yunnan, China	MK270569	Yunnan
FG035	*I. mors-panacis*	*P. notoginseng*	Yunnan, China	MK270571	Yunnan
FG036	*I. mors-panacis*	*P. notoginseng*	Yunnan, China	MK270572	Yunnan
JYHHC-6	*I. mors-panacis*	*P. ginseng*	Northeast, China	MN101774	Northeast
JTJJA-1	*I. mors-panacis*	*P. ginseng*	Northeast, China	MN101775	Northeast
JBJMJ-c	*I. mors-panacis*	*P. ginseng*	Northeast, China	MN101776	Northeast
JBFQY-4	*I. mors-panacis*	*P. ginseng*	Northeast, China	MN101777	Northeast
SQPC-3	*I. mors-panacis*	*P. notoginseng*	Yunnan, China	KU219938	Yunnan
SQPC-4	*I. mors-panacis*	*P. notoginseng*	Yunnan, China	KU219939	Yunnan
XGD1b1-2	*I. mors-panacis*	*P. quinquefolius*	Shandong, China		Shandong
OZTB7-1	*I. mors-panacis*	*P. quinquefolius*	Shandong, China		Shandong
OZTB8-4	*I. mors-panacis*	*P. quinquefolius*	Shandong, China		Shandong
OZT1-1-1	*I. mors-panacis*	*P. quinquefolius*	Shandong, China		Shandong
OZT1-1-2	*I. mors-panacis*	*P. quinquefolius*	Shandong, China		Shandong
OZT1-1-3	*I. mors-panacis*	*P. quinquefolius*	Shandong, China		Shandong
OZT1-2-1	*I. mors-panacis*	*P. quinquefolius*	Shandong, China		Shandong
OZT1-2-2	*I. mors-panacis*	*P. quinquefolius*	Shandong, China		Shandong
OZT1-3-4	*I. mors-panacis*	*P. quinquefolius*	Shandong, China		Shandong
OZT1-5-2	*I. mors-panacis*	*P. quinquefolius*	Shandong, China		Shandong
OZT1-5-4	*I. mors-panacis*	*P. quinquefolius*	Shandong, China		Shandong
OSC2-1-2	*I. mors-panacis*	*P. quinquefolius*	Shandong, China		Shandong
OSC2-2-2	*I. mors-panacis*	*P. quinquefolius*	Shandong, China		Shandong
OSC2-3-2	*I. mors-panacis*	*P. quinquefolius*	Shandong, China		Shandong

OSC2-5-2	*I. mors-panacis*	*P. quinquefolius*	Shandong, China		Shandong
OSC2-6-1	*I. mors-panacis*	*P. quinquefolius*	Shandong, China		Shandong
OZTB7-3	*I. mors-panacis*	*P. quinquefolius*	Shandong, China		Shandong
OQT11-3	*I. mors-panacis*	*P. quinquefolius*	Shandong, China		Shandong
OZTB2-1	*I. mors-panacis*	*P. quinquefolius*	Shandong, China		Shandong
OZTB3-3	*I. mors-panacis*	*P. quinquefolius*	Shandong, China		Shandong
OZTB3-4	*I. mors-panacis*	*P. quinquefolius*	Shandong, China		Shandong
OZT1-3-3	*I. mors-panacis*	*P. quinquefolius*	Shandong, China		Shandong
1-2-1	*I. mors-panacis*	*P. quinquefolius*	Shandong, China		Shandong
1-2-2	*I. mors-panacis*	*P. quinquefolius*	Shandong, China		Shandong
2-2-2	*I. mors-panacis*	*P. quinquefolius*	Shandong, China		Shandong
3-1-1	*I. mors-panacis*	*P. quinquefolius*	Shandong, China		Shandong
3-2-1	*I. mors-panacis*	*P. quinquefolius*	Shandong, China		Shandong
4-3-1	*I. mors-panacis*	*P. quinquefolius*	Shandong, China		Shandong
5-2-1	*I. mors-panacis*	*P. quinquefolius*	Shandong, China		Shandong
FOH9-3	*I. mors-panacis*	*P. quinquefolius*	Shandong, China	MN833110	Shandong
S10-1	*I. mors-panacis*	*P. quinquefolius*	Shandong, China		Shandong
12-3-1	*I. robusta*	*P. quinquefolius*	Shandong, China	MN833112	Shandong
OZTB1-2	*I. robusta*	*P. quinquefolius*	Shandong, China	MN833113	Shandong
SX1	*I. mors-panacis*	*P. notoginseng*	Yunnan, China		Yunnan
SX2	*I. mors-panacis*	*P. notoginseng*	Yunnan, China		Yunnan
SX4	*I. mors-panacis*	*P. notoginseng*	Yunnan, China		Yunnan
SX5	*I. mors-panacis*	*P. notoginseng*	Yunnan, China		Yunnan
SZ1	*I. mors-panacis*	*P. notoginseng*	Yunnan, China		Yunnan
SZ2	*I. mors-panacis*	*P. notoginseng*	Yunnan, China		Yunnan
SZ3	*I. mors-panacis*	*P. notoginseng*	Yunnan, China		Yunnan
SZ5	*I. mors-panacis*	*P. notoginseng*	Yunnan, China		Yunnan
SS3	*I. mors-panacis*	*P. notoginseng*	Yunnan, China		Yunnan
SS4	*I. mors-panacis*	*P. notoginseng*	Yunnan, China		Yunnan
SS5	*I. mors-panacis*	*P. notoginseng*	Yunnan, China		Yunnan
